# Near‐Infrared Carbonized Polymer Dots for NIR‐II Bioimaging

**DOI:** 10.1002/advs.202203474

**Published:** 2022-09-01

**Authors:** Tianyang Han, Yajun Wang, Shengjie Ma, Mengfei Li, Ningning Zhu, Songyuan Tao, Jiajun Xu, Bin Sun, Yunlong Jia, Yuewei Zhang, Shoujun Zhu, Bai Yang

**Affiliations:** ^1^ State Key Laboratory of Supramolecular Structure and Materials College of Chemistry Jilin University Changchun 130012 P. R. China; ^2^ Joint Laboratory of Opto‐Functional Theranostics in Medicine and Chemistry The First Hospital of Jilin University Changchun 130021 P. R. China; ^3^ Department of Gastrointestinal Surgery The First Hospital of Jilin University Changchun 130021 P. R. China; ^4^ School of Chemistry and Pharmaceutical Engineering Jilin Institute of Chemical Technology Jilin 132022 P. R. China

**Keywords:** carbonized polymer dots, in vivo imaging of colitis, molecular state, near‐infrared‐II bioimaging, near‐infrared ‐II angiography

## Abstract

Carbon dots (CDs) or carbonized polymer dots (CPDs) are an emerging class of optical materials that have exceptional applications in optoelectronic devices, catalysis, detection, and bioimaging. Although cell studies of CPDs have produced impressive results, in vivo imaging requires available CPDs to fluoresce in the near‐infrared‐II (NIR‐II) window (1000−1700 nm). Here, a two‐step bottom‐up strategy is developed to synthesize NIR‐CPDs that provide bright emissions in both NIR‐I and NIR‐II transparent imaging windows. The designed strategy includes a hydrothermal reaction to form a stable carbon core with aldehyde groups, followed by the Knoevenagel reaction to tether the molecular emission centers. This procedure is labor‐saving, cost‐efficient, and produces a high yield. The NIR‐CPDs enable high‐performance NIR‐II angiography and real‐time imaging of the disease degree of colitis noninvasively. This technology may therefore provide a next‐generation synthesis strategy for CPDs with rational molecular engineering that can accurately tune the absorption/emission properties of NIR‐emissive CPDs.

## Graphical abstract

Carbonized polymer dots (CPDs) are remarkable optical materials with low preparation cost, high biocompatibility, and excellent optical properties. The emission wavelengths of current CPDs are limited in the visible to NIR‐I range, impeding their applications in the transparent NIR‐II window. A new series of NIR‐CPDs is reported through a two‐step hydrothermal/Knoevenagel reaction with adjustable absorption/emission wavelength for NIR‐II bioimaging.

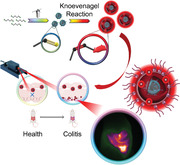

## 1 Introduction

Despite the considerable advantages of carbonized polymer dots (CPDs) in the fields of fluorescent and phosphorescent materials,^[^ ^1^ ^]^ the relatively short emission wavelength remains a major obstacle in promoting them for in vivo disease detection and imaging‐guided surgery. Currently, robust synthesis protocols for CPDs have produced many bright CPDs with emission wavelengths in the visible to near‐infrared (NIR) region (400−900 nm).^[^ ^2^ ^]^ However, owing to tissue autofluorescence and scattering in the region, these CPDs fail to provide high‐performance in vivo imaging for disease detection with high contrast and deep penetration depth.

NIR‐II (originally defined as 1000−1700 nm, and recently perfected as 900−1880 nm^[^ ^3^ ^]^ or 1000−3000 nm;^[^ ^4^ ^]^ also termed shortwave infrared or SWIR) transparent fluorescence imaging can partially solve this problem, benefitting from the much diminished tissue autofluorescence and suppressed photon scattering.^[^ ^5^ ^]^ Thus, substantial efforts have been made to enrich the library of NIR‐II fluorophores, with typical examples including carbon nanotubes, quantum dots, lanthanide‐doped nanoparticles, conjugated polymers, and small molecules.^[^ ^6^ ^]^ Because CPDs constitute a class of new fluorophores with convenient synthesis procedures and efficiently tunable chemical structures, exploiting CPDs with bright NIR emission as a standard‐of‐use imaging agent in the NIR‐II imaging window will likely usher in a new era of disease detection and surgical visualization.

Although the hydrothermal reaction has been widely used to synthesize CPDs,^[^ ^7^ ^]^ few reports have focused on rational molecular engineering on the surface of hydrothermal CPDs to accurately tune the emission centers.^[^ ^2^, ^8^ ^]^ Investigating this point is likely essential to enrich the molecular state and develop CPDs with improved NIR‐emissive properties. Here, we investigated and developed a series of NIR‐CPDs through a two‐step hydrothermal/Knoevenagel method with tunable emission centers fluorescing in both NIR‐I and NIR‐II windows. The NIR‐CPDs possessed considerable NIR‐II brightness in water solution and can be further improved by using albumin or an amphiphilic polymer encapsulation/cocktail.^[^ ^9^ ^]^ NIR‐CPDs displayed very low cytotoxicity and good biocompatibility, which enables them to be used for high‐performance NIR‐II angiography and real‐time assessment of disease severity in colitis.^[^ ^10^ ^]^ We not only provide an efficient strategy to synthesize NIR‐emission CPDs but also perform non‐invasive real‐time in vivo imaging to further promote their preclinical/clinical applications.

## 2 Results

### 2.1 Design and Synthesis of NIR‐CPDs through A Two‐Step Route with Adjustable Absorption/Emission Wavelength

**Figure 1:**
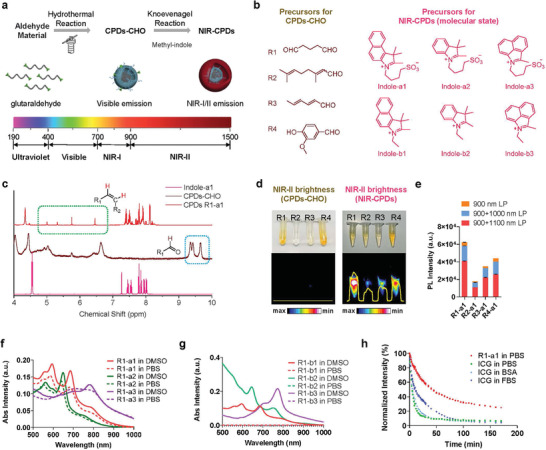
Preparation of NIR‐CPDs through two‐step hydrothermal/Knoevenagel reaction. a) Schematic synthesis process of CPDs‐CHO and NIR‐CPDs. b) Alternative precursors for CPDs‐CHO and NIR‐CPDs, respectively. c) ^1^H NMR of indole‐a1, R1‐CHO, and CPDs R1‐a1. d) Photograph and NIR‐II brightness (over 900 nm) of CPDs‐CHO and NIR‐CPDs, respectively. e) Statistics of the fluorescence signal (heights of histogram) of NIR‐CPDs under different long‐pass filters (LP) with different carbon precursors. f) Absorption spectra of a‐serial NIR‐CPDs in DMSO and PBS buffers. g) Absorption spectra of b‐serial NIR‐CPDs. These NIR‐CPDs are not water‐soluble; thus, there is no detectable signal in PBS buffer. h) Photostability of NIR‐CPDs R1‐a1 in PBS and ICG with equivalent OD at 808 nm in PBS, BSA (50 mg mL^−1^), and FBS (Imaging condition: 808 nm laser excitation with 63.2 mW cm^−2^ power density, 900 nm long‐pass filters). All data are expressed as mean ± SD.

We designed a two‐step method that includes both hydrothermal and Knoevenagel reactions to synthesize NIR‐CPDs (**Figure** **1**a). During the first step, CPDs‐CHO were easily synthesized through the hydrothermal method by using inexpensive and readily available aldehyde precursors. During the second step, the NIR‐CPDs were synthesized via the Knoevenagel reaction between CPDs‐CHO and a set of indole derivatives (Figure 1b). The indole derivatives can form a strong molecular state emission center after covalent binding on the surface of conjugated CPDs‐CHO. The NIR‐CPDs were purified through dialysis against deionized (DI) water.^[^ ^1e^ ^]^ By tuning the precursors for both steps, we accessed a diverse set of NIR‐CPDs, named NIR‐CPDs Rx‐ay or Rx‐by (where *x* represents 1, 2, 3, and 4, and *y* represents 1, 2, and 3). Compared with the one‐step hydrothermal route with the same precursors, NIR‐CPDs prepared via the proposed two‐step method provided a much higher production yield and NIR‐II brightness (Figure S1a,b, Supporting Information). The NIR‐CPDs also exhibited a slightly longer emission peak (Figure S1c, Supporting Information) and a much lower cost than the commercial dye, indocyanine green (ICG; this dye was selected for comparison because it has the same side chains as our NIR‐CPDs) (Figure S1d,e, Supporting Information).

Further characterization was necessary to determine the chemical structure of the CPDs‐CHO and NIR‐CPDs. To verify that the indole derivative was combined through a covalent bond during the second step, ^1^H Nuclear magnetic resonance (NMR) of indole‐a1, CPDs‐CHO, and NIR‐CPDs was performed (Figure 1c). The active hydrogen peak for aldehyde groups at *δ* 9.36−9.67 disappeared from CPDs to NIR‐CPDs, and a new peak for the cis‐conjugate double bond at *δ* 6.46−5.01 (*J* = 4.00 Hz < 12.00 Hz) appeared for NIR‐CPDs, indicating that the new conjugate double bond was formed by the Knoevenagel reaction during the second step. The hydrogen peak in the aromatic region of the indole structure (*δ* > 7.00) also proved that the indole groups were connected with CPDs‐CHO. Moreover, the NIR‐II brightness signal was only observed for NIR‐CPDs and no detectable signal was observed for their CPDs‐CHO intermediates (Figure 1d,e), indicating that the NIR‐II signal was derived from the formed molecular state. NIR‐CPDs prepared from R1 (glutaraldehyde) exhibited higher NIR‐II brightness than the other three aldehyde precursors; we thus chose R1 for synthesizing CPDs‐CHO in the rest of the study.

For CPDs with the molecular state as the emission mechanism, the photoluminescence can be flexibly tuned by the connected molecular center. As shown in Figure 1b, we chose six types of methyl‐indole derivatives (three of which are soluble in water) to test the NIR‐II properties of NIR‐CPDs. NIR‐CPDs with different molecular centers exhibited tunable absorption peaks from 639 to 781 nm and the corresponding emission peaks ranging from 907 to 927 nm (Figure 1f,g, Table S1, Supporting Information). Notably, some NIR‐CPDs exhibited a relatively large Stokes shift (>100 nm). The NIR fluorescence of NIR‐CPDs can even be excited through visible light (485 nm) (Figure S2, Supporting Information).

The covalent combination of indole derivatives also changed the water solubility of the CPDs. For a‐serial NIR‐CPDs with sulfonated groups, the optical properties were consistent in both dimethyl sulfoxide (DMSO) and water solutions (Figure 1f). Conversely, there was no signal for b‐serial NIR‐CPDs dispersed in water (Figure 1g). All the NIR‐CPDs show remarkable fluorescence intensity in the NIR region from 900 to 1200 nm (Figures S2, S3a,b, Supporting Information), and the emissions of R1‐a3 and R1‐b3 were still detectable up to 1500 nm.

The carbon core structure of the NIR‐CPDs presumably improved light absorption and photostability. The continuous conjugated double bond structure of traditional cyanine dyes is generally weak and unstable.^[^ ^11^ ^]^ Conversely, CPDs‐CHO provides a crosslinked conjugated network, which can substantially improve the photostability of NIR‐CPDs. The results verified that NIR‐CPDs exhibited much‐improved photostability compared with commercially available cyanine dyes (ICG and IR‐783) in either phosphate‐buffered saline (PBS), bovine serum albumin (BSA, 50 mg mL^−1^), or fetal bovine serum (FBS) (Figure 1h, Figure S3c, Supporting Information). Surprisingly, the photostability of some NIR‐CPDs is further enhanced when combined with BSA or FBS (Figure S3d–i, Supporting Information). Optical information in terms of *λ* (Abs/Em), quantum yields (QYs), and extinction coefficient is provided in Table S1 and Figures S4, S5, Supporting Information. Collectively, the rationally tunable molecular state provides great opportunities for tailoring the structures to match other desired photoluminescence and applications.

### 2.2 Characterization of CPDs‐CHO and their Optimization for NIR‐CPDs

**Figure 2:**
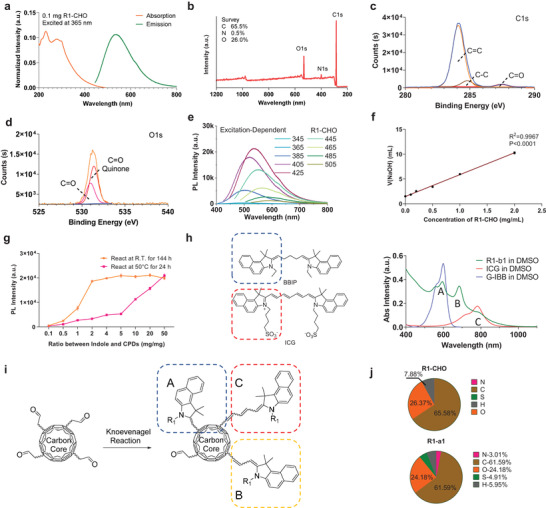
Structure characterization of CPDs‐CHO and NIR‐CPDs. a) Absorption spectra and emission spectra of R1‐CHO. b) XPS survey spectra of CPDs‐CHO. c) The high‐resolution C1s XPS and d) O1s XPS spectra of CHO‐CPDs. e) Photoluminescence emission profiles of R1‐a1 under the selected excitation wavelengths. f) The number of aldehyde groups on CPDs‐CHO was determined via hydroxylamine hydrochloride and NaOH titration. g) Optimal reaction ratio between CPDs‐CHO and indole precursor was screened through two typical reaction temperatures (R.T. and 50 °C). h) Comparison of suggested molecular centers and absorption spectra of R1‐b1, ICG, and BBIP). i) Schematic molecular state emission centers (A, B, and C) of NIR‐CPDs. j) Element analysis of CPDs R1‐CHO and NIR‐CPDs R1‐a1. All data are expressed as mean ± SD. For normally distributed data sets with equal variances, one‐way ANOVA testing followed by a Tukey post‐hoc test was carried out across groups.

We systematically investigated the chemical structure and optical properties of CPDs‐CHO, which is essential for manufacturing the final NIR‐CPDs. The CPDs‐CHO was synthesized via the one‐step hydrothermal method using 1.0 g fatty aldehyde dissolved in 8 mL of 50% ethanol‐water mixed solution (see the Experimental Section in Supporting Information for details).^[^ ^12^ ^]^ Absorption spectra of R1‐CHO exhibited stronger absorption in K and E2 bands compared with R1 precursor, indicating the formation of conjugated polyene structure. Notably, CPDs‐CHO from R1 (glutaraldehyde) showed the largest increase among all the precursors (Figure S6a,b, Supporting Information). The CPDs‐CHO displayed an absorption peak in the UV region and an emission peak at 539 nm (**Figure** **2**a).

The chemical structure of CPDs‐CHO was further explored using X‐ray photoelectron spectroscopy (XPS). The survey spectrum (Figure 2b) indicated that CPDs‐CHO mainly consists of carbon (C, 91.5%) and oxygen (O, 8.0%), with a minute amount of nitrogen (N, 0.5%). The high‐resolution C1s spectrum (Figure 2c) revealed three types of carbon in CPDs‐CHO, including C=C (284.1 eV), C—C (284.8 eV), and C=O (287.5 eV), while the C=O groups (532.3 eV) were also detected in the O1s spectrum (Figure 2d). In addition, the quinone C=O (531.0 eV) was fitted via simulation in the analysis software, which is consistent with the above hypothesized conjugated aldehyde groups in CPDs‐CHO. Fourier transform infrared spectroscopy (FTIR) was used to determine the surface functional groups on the surfaces of CPDs‐CHO and NIR‐CPDs. The characteristic peaks of the aldehyde groups at 1725 cm^−1^ in the FTIR spectrum of CPDs‐CHO (Figure S6c, Supporting Information) changed/disappeared in the FTIR spectrum of the NIR‐CPDs.

Next, the morphology of CPDs‐CHO was characterized via transmission electron microscopy (TEM), wherein the CPDs‐CHO from precursor R1 showed a nearly spherical shape with a diameter of 2–4 nm (Figure S6d, Supporting Information). The average weight of CPDs‐CHO tested using the MALDI‐TOF mass spectrometer was ≈1000 (Figure S6e, Supporting Information). The average weight of NIR‐CPDs was characterized by gel permeation chromatography (GPC), and the weighted average molecular weight (MW) was approximately determined to be 2936 (numerically average molecular weight = 2921) (Figure S6f, Supporting Information).

To further understand the formation mechanism and internal structure of CPDs‐CHO. We subjected glutaraldehyde to polymerization or hydrothermal reactions under different reaction conditions and detected their structural changes by ^1^H NMR (Figure S6g, Supporting Information). With the intensification of reaction conditions, the peaks of large chemical shifts in different reaction conditions begin to increase. While the peaks of the aliphatic structures do not change significantly in all reaction systems, this indicates that after a violent hydrothermal reaction, CPDs‐CHO still retains a large number of aliphatic chain structures. Considering that the hydration radius measured in DLS is much larger than that measured in TEM (Figure S6d, Supporting Information), the existence of polymer structure in CPDs‐CHO is reasonable.

Excitation‐dependent behavior was observed in all CPDs‐CHO, which is consistent with previous reports (Figure 2e, Figure S7a–c, Supporting Information). The emission peak of CPDs‐CHO (R1‐CHO) changed from 475 to 577 nm when the corresponding excitation wavelength changed from 345 to 505 nm. Conversely, the excitation‐dependent behavior disappeared after modification with indole derivatives (Figure S7d–g, Supporting Information). This phenomenon further verified that the emission center of our NIR‐CPDs was derived from the molecular state, while the carbon core served as a type of light absorption center (antenna effect).^[^ ^13^ ^]^

For CPDs‐CHO, the number of aldehyde groups is very important for further modification of indole derivatives. The concentration of aldehyde groups on CPDs‐CHO was determined using the hydroxylamine hydrochloride titration method (Figure 2f).^[^ ^14^ ^]^ The plotted results verify that the concentration of aldehyde groups was 4.5 µmol mg^−1^ (see the Experimental Section in the Supporting Information for details). Different concentrations of indole‐a1 were mixed with R1‐CHO to optimize the reaction ratio between CPDs‐CHO and methyl‐indole (Figure 2g).

To further study the chemical structure of the molecular state emission center of NIR‐CPDs, we designed a small molecule (1Z,5Z)‐1,5‐bis(3‐ethyl‐1,1‐dimethyl‐1,3‐dihydro‐2H‐benzo[e]indol‐2‐ylidene)pentane (BBIP) to compare the position of the absorption peak of the luminescence structure through the Knoevenagel reaction between glutaraldehyde and indole b1 (Figure 2h). From the characteristic absorption peaks of absorption spectra, we speculate that there are three main luminescent centers (A, B, and C) in NIR‐CPDs, which are possibly generated by the reaction of conjugate aldehyde groups of different lengths and chromophores (Figure 2i). In addition, the contents of N and S elements were monitored from CPDs‐CHO to NIR‐CPDs through element analysis (Figure 2j). The results indicated that the percentage of N and S elements remarkably increased from CPDs‐CHO to NIR‐CPDs, again verifying the successful connection between indole derivatives and CPDs‐CHO.

Because of the low quantum efficiency of silicon detectors,^[^ ^15^ ^]^ previously reported CPDs may have reasonable emissions in the NIR region. We synthesized three typical CPDs from citric acid and tunable amine precursors and compared their NIR‐I/II brightness with our NIR‐CPDs (Figure S8, Supporting Information).^[^ ^16^ ^]^ The results indicated that a very weak NIR signal was detected for these previously reported CPDs,^[^ ^1^, ^17^ ^]^ while our NIR‐CPDs showed remarkable NIR‐I/II signals.

### 2.3 Biosafety Assessment and High‐Performance NIR‐II Angiography of NIR‐CPDs

**Figure 3:**
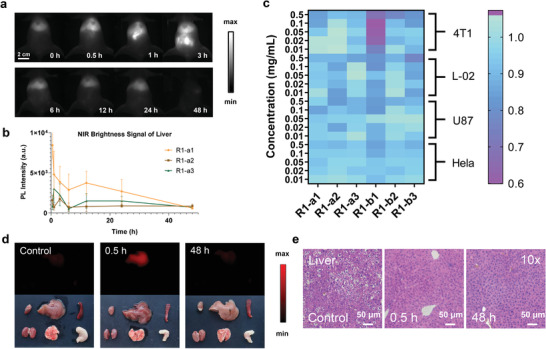
Biosafety assessment of NIR‐CPDs. a) Selected NIR‐II imaging of mice in the supine position after intravenous administration of NIR‐CPDs taken as a function of the injected period (injection dosage, 0.1 mg mL^−1^, 200 µL; imaging condition: 808 nm laser excitation with 63.2 mW cm^−2^ power density, 900 + 1000 nm long‐pass filters). b) NIR‐II fluorescence signal of the liver for NIR‐CPDs over the relevant period. c) Cell viability of NIR‐CPDs using 4T1, L‐02, U87, and HeLa cell lines through MTT commercial kit. d) NIR‐II fluorescence images (upper) and photographs (lower) of organ anatomy (heart, liver, spleen, kidney, lung, and stomach) from the control group and NIR‐CPDs administered cohort at 0.5 and 48 h. e) Histological evaluation of liver after intravenous administration of NIR‐CPDs. All data are expressed as mean ± SD. For normally distributed data sets with equal variances, one‐way ANOVA testing followed by a Tukey post‐hoc test was carried out across groups.

Although CPDs have been repeatedly reported to have good biocompatibility,^[^ ^1^, ^2^, ^17^, ^18^ ^]^ we are still interested in assessing the biotoxicity of our NIR‐CPDs before subjecting them to in vivo applications. Both a‐serial and b‐serial NIR‐CPDs (injection dosage: 0.1 mg mL^−1^, 200 µL) were intravenously (i.v.) injected into mice to observe their metabolism behaviors. All the NIR‐CPDs were excreted from the mice via feces, indicating a classic hepatobiliary clearance pathway (**Figure** **3**a, Figure S9, Supporting Information). Plotting the NIR‐II brightness signal of the liver for a‐serial NIR‐CPDs (which are water‐soluble) against post‐injection time points resulted in an obvious signal depletion (Figure 3b). To further evaluate the biosafety of NIR‐CPDs, cell viability was tested through four types of cell lines: 4T1, L‐02, U87, and HeLa. The results indicated that all a‐serial NIR‐CPDs exhibited very low cytotoxicity, while some of the b‐serial NIR‐CPDs exhibited low cell accumulation or cytotoxicity (Figure 3c). In addition, mice organs (heart, liver, spleen, kidney, lung, and stomach) at two typical metabolic time points (0.5 and 48 h) were completely harvested, coupled with the same organs from the saline‐injected control group, and subjected to NIR‐II imaging to estimate the NIR‐CPDs accumulation and excretion rate (Figure 3d). As shown from the imaging results, the NIR‐II signal was only observed in the liver from the cohort at the 0.5 h time point, while all the main organs eliminated the signal at 48 h post‐injection. Pathology analysis of liver from saline and NIR‐CPDs administered cohorts of mice by hematoxylin and eosin (H&E) staining also verified that there was no obvious abnormal morphology (Figure 3e). Collectively, NIR‐CPDs did not cause any toxic reactions both in vitro and in vivo, primed for NIR‐II bioimaging of desired biological events.

Cyanine dyes are widely reported to be encapsulated by either exogenous or endogenous albumin to form stable complexes for deep tissue bioimaging.^[^ ^19^ ^]^ The cyanine dye/albumin complex can efficiently reduce non‐radiative processes by providing physical restriction for the cyanine dyes and providing electron‐donor interaction, yielding improved twisted intramolecular charge transfer (TICT) effect and enhancing NIR‐II tail emission.^[^ ^5^, ^19^, ^20^ ^]^ To this end, we investigated whether our NIR‐CPDs can benefit from this complex strategy. Compared with NIR‐CPDs R1‐a1 in PBS buffer, our NIR‐CPDs showed an ≈sixfold brightness enhancement when mixed with BSA (50 mg mL^−1^) and ≈12‐fold improvement when mixed with FBS (Figure S10a, Supporting Information). All the a‐serial NIR‐CPDs displayed brightness enhancement after being mixed with BSA under different sub‐NIR‐II windows from 900 to 1400 nm (Figure S10b, Supporting Information). In addition, there were no obvious differences in brightness enhancement between heating at 37 and 50 °C for 4 h (Figure S10c, Supporting Information), indicating that there were no energy‐dependent complexation processes between NIR‐CPDs and albumin. Increasing the ratio of albumin to NIR‐CPDs (0.1 mg mL^−1^) resulted in brightness enhancement (Figure S10d, Supporting Information). Protein electrophoresis of free NIR‐CPDs and their mixture with BSA after heating indicated that there was no covalent binding between NIR‐CPDs and BSA (Figure S10e,f, Supporting Information). Taken together, although the complexation between NIR‐CPDs and albumin was not very stable, this albumin‐chaperoned strategy substantially improved the NIR‐II brightness of NIR‐CPDs.

There is a high concentration of albumin (≈35−50 mg mL^−1^) in the blood plasma of living organisms. Thus, NIR‐CPDs will hitchhike endogenous albumin in vivo to yield high‐performance NIR‐II angiography. We performed mice brain and hindlimb vessel imaging after intravenous injection of NIR‐CPDs R1‐a1 (3 mg mL^−1^ in PBS, 200 µL). Whole‐body imaging indicated that NIR‐CPDs indeed provided remarkable imaging brightness and signal‐to‐noise (S/B) ratio at relatively low power densities and exposure time under 1000, 1100, 1200, and 1300 nm NIR‐II windows (Figure S11a, Supporting Information). Compared with the NIR‐I imaging (850−1000 nm), NIR‐CPDs provided significantly improved imaging quality and a better S/B ratio for both hindlimb and brain vessels (Figure S11b–e, Supporting Information). The NIR‐II angiographic time window of administration for NIR‐CPDs can last for at least 10 min with the desired imaging contrast. Considering their improved photostability, NIR‐CPDs have the potential to further broaden the time window for NIR‐II angiography.

### 2.4 Encapsulation of NIR‐CPDs Further Improved the NIR‐II Imaging Quality

**Figure 4:**
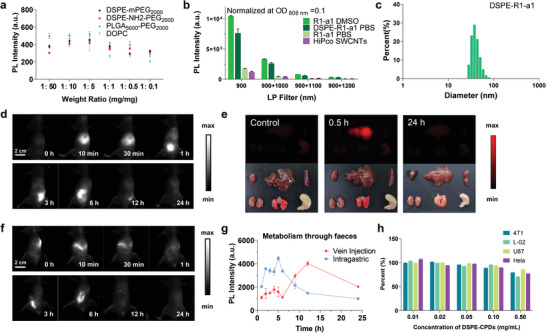
NIR‐CPDs encapsulated by DSPE‐mPEG‐2000 micelles provided the improved water solubility and NIR‐II brightness. a) NIR‐II fluorescence intensity of NIR‐CPDs micelles/cocktails by using four commercial amphiphilic copolymers with different ratios between CPDs and commercial amphiphilic copolymers (dioleoyl‐snglycero‐3‐phosphocholine, DPCO). b) Fluorescence intensity of NIR‐CPDs R1‐a1 in DMSO, PBS, and DSPE‐R1‐a1 in PBS compared with HiPco SWCNTs. c) Dynamic light scattering (DLS) analysis of DSPE‐R1‐a1 (0.1 mg mL^−1^) in PBS. d) NIR‐II fluorescence time points of mice in the supine position after intravenous administration of DSPE‐R1‐a1 taken as a function of the injected time (injection dosage: 0.1 mg mL^−1^, 200 µL). e) NIR‐II fluorescence imaging and photograph of organ anatomy (heart, liver, spleen, kidney, lung, and stomach) from control and DSPE‐R1‐a1 injected cohorts. f) NIR‐II fluorescence time points of mice in the supine position after intragastric administration of DSPE‐R1‐a1 (0.1 mg mL^−1^, 200 µL). g) Fluorescence intensity of the collected feces after intragastric and intravenous injection of DSPE‐R1‐a1 at relevant time points. h) Cell viability of DSPE‐R1‐a1 using 4T1, L‐02, U87, and HeLa cell lines. All data are expressed as mean ± SD. For normally distributed data sets with equal variances, one‐way ANOVA testing followed by a Tukey post‐hoc test was carried out across groups.

After proving the high photostability and hepatobiliary clearance pathway of NIR‐CPDs, we performed colitis imaging to assess their NIR‐II imaging performance. Although free NIR‐CPDs provided reasonable NIR‐II imaging ability, we found that amphiphilic polymers could further improve their imaging quality by enhancing their NIR‐II brightness and in vivo accumulation (especially for colitis imaging). After screening several commercial amphiphilic polymers with lipid groups for encapsulation of NIR‐CPDs, we determined the optimal encapsulation ratio between NIR‐CPDs and amphiphilic polymers to be 1:5 (**Figure** **4**a). DSPE‐mPEG2000 was finally selected to manufacture the encapsulated complex/cocktail, named DSPE‐CPDs (or DSPE‐R1‐a1). The as‐prepared DSPE‐R1‐a1 achieved ≈fourfold brightness enhancement compared to free R1‐a1 in PBS (Figure 4b). The DSPE‐R1‐a1 exhibited an average hydrodynamic diameter of 30–40 nm when detected by dynamic light scattering (DLS) (Figure 4c).

To ensure that the complex does not cause additional potential biotoxicity, the biological metabolism of DSPE‐R1‐a1 was evaluated after intravenous injection of DSPE‐R1‐a1. The results indicated that DSPE‐R1‐a1 also displayed a hepatobiliary‐intestines‐feces clearance pathway, while the excretion rate was relatively faster than that of free R1‐a1 (Figure 4d). A consistent conclusion was also suggested by organ distribution at both 0.5 and 24 h post‐injection time points (Figure 4e). Considering that oral administration is more suitable for clinical practice than intravenous administration, particularly for gastrointestinal diseases, we further performed oral administration of DSPE‐R1‐a1 in the mice and observed metabolic behavior followed by intragastric administration (Figure 4f). In this case, DSPE‐R1‐a1 migrated along the gastrointestinal tract and was eventually excreted via feces. By collecting mice feces and plotting the signal of feces against post‐injection time points for both intravenous and intragastric administration cohorts of mice, DSPE‐CPDs by intragastric administration were found to display a much faster excretion rate (Figure 4g). In addition, the cytotoxicity of DSPE‐R1‐a1 was further evaluated and extremely high cell viability was observed even up to 0.5 mg mL^−1^ administered dosage (Figure 4h).

### 2.5 DSPE‐CPDs Provided High‐Contrast and Deep‐Penetration NIR‐II Colitis Imaging

**Figure 5:**
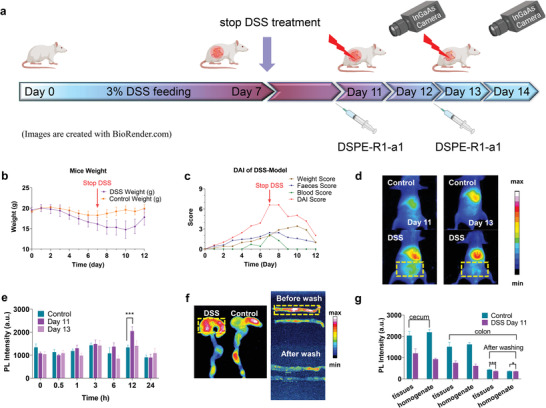
NIR‐CPDs provided NIR‐II imaging of acute colitis with high spatiotemporal resolution. a) Schematic diagram and experiment timeline of the assessment of disease severity in acute colitis using DSPE‐R1‐a1 (DSS, dextran sulfate sodium salt). b) Differences in body weight of control and DSS‐treated cohorts of mice. c) Disease activity index (DAI) score of DSS‐treated mice. d) Representative NIR‐II images were acquired 12 h after intravenous administration of DSPE‐R1‐a1 using DSS‐treated mice on days 11 and 13, respectively (injection dosage: 0.1 mg mL^−1^, 200 µL). e) NIR‐II fluorescence signal of the abdomen (the statistical ROI area is shown in (d)) as a function of post‐injection time points for control, DSS treatment for 11 and 13 d cohorts of mice. f) NIR‐II fluorescence imaging of colon and cecum after anatomy at 12 h. g) The signals comparison of colon and cecum from control and DSS treatment for 11 d cohort of mice. All data are expressed as mean ± SD. For normally distributed data sets with equal variances, one‐way ANOVA testing followed by a Tukey post‐hoc test was carried out across groups. Significance was defined as * *p* < 0.05, **p < 0.01, ***p < 0.001.

Following the reported protocol,^[^ ^21^ ^]^ the colitis model was established through oral administration of dextran sulfate sodium salt (DSS). Acute colitis was induced in mice by administering drinking water containing 3% DSS for seven consecutive days (see the Experimental section for details). We first performed the imaging experiment on the protocol‐suggested days 11 and 13 after DSS treatment (see timeline in **Figure** **5**a). During the establishment of the colitis model, the loss in weight of the mice cohort was observed over time and it gradually recovered after DSS administration was stopped on day 7 (Figure 5b). This is consistent with the previously reported phenomenon of acute colitis.^[^ ^21^, ^22^ ^]^ The inflammatory model was further assessed using the classical disease activity index (DAI), including animal weight loss, fecal stickiness, and fecal bleeding. With DSS administration, the disease degree of the mice gradually deepened and reached its maximum on days 7 and 8. Some of the mice died at this stage (≈5%); however, the remaining mice began to recover rapidly following this stage (Figure 5c).

On days 11 and 13 after DSS treatment, DSPE‐R1‐a1 was intravenously administered to mice with colitis; simultaneously, the control group was administered only saline. After injection, the intestinal shape was clearly visualized and the biodistribution of DSPE‐R1‐a1 over any position was determined by analyzing the NIR‐II signal (>1000 nm) at the region(s) of interest (ROI) (Figure S12a, Supporting Information). Compared to the imaging results taken at the typical 12 h time point, the cohorts of mice injected with DSPE‐CPDs displayed significantly enhanced NIR‐II signals from the abdominal intestinal position compared with the control group (Figure 5d, Figure S12b, Supporting Information). Plotting the abdominal signals against post‐injection time points confirmed that the maximum accumulation of DSPE‐CPDs indeed occurred at 12 h post‐injection (Figure 5e). The accumulation of DSPE‐R1‐a1 at the inflammatory site was further confirmed via ex vivo dissection of the cecum/colon portion. After dissection, PBS was used to thoroughly rinse the lateral cut colon, and the average signal intensity of the cecum, colon, and washed cecum/colon was recorded (Figure 5f). We further homogenized these organs and plotted the NIR‐II signals for both intact and the homogenate of cecum/colon, and the results showed statistical differences for all tested groups (Figure 5g). To verify that DSPE‐mPEG2000 encapsulation is essential for efficient colitis imaging, R1‐a1 and DSPE‐R1‐a1 were separately administered to mice with colitis through the tail vein on day 11 of the colitis model; here, saline‐injected mice with colitis were used as a control group (Figure S12c, Supporting Information). R1‐a1 showed no accumulation in the inflammatory bowel in both in vivo abdominal statistics (Figure S12d, Supporting Information) and ex vivo dissected cecum (Figure S12e, Supporting Information), indicating that the DSPE‐mPEG2000 encapsulation remarkably improved the colitis imaging ability of our NIR‐CPDs.

**Figure 6:**
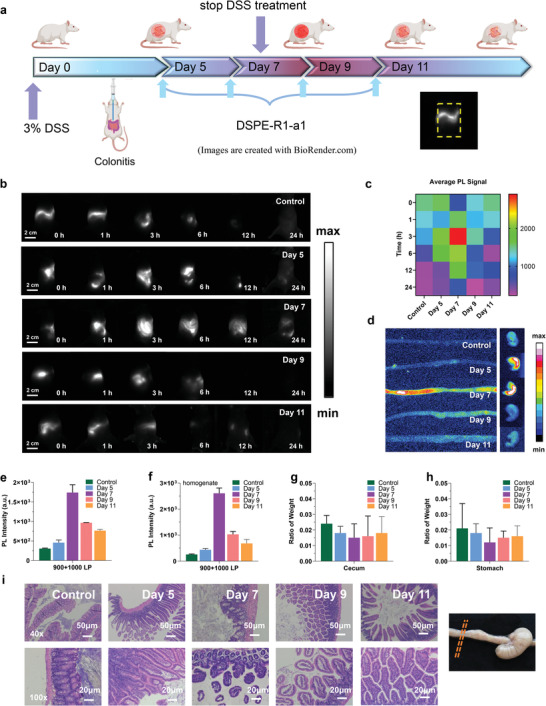
NIR‐II imaging and evaluation of colitis upon oral administration of NIR‐CPDs. a) Experiment timeline of the assessment of disease severity in acute colitis modal through DSPE‐R1‐a1. b) NIR‐II fluorescence imaging of DSS‐treated mice on days 5, 7, 9, and 11 in the supine position after intragastric administration of DSPE‐R1‐a1 (injection dosage: 0.1 mg mL^−1^, 200 µL). c) Average abdominal fluorescence signal statistics for both control and DSS‐treated groups at relevant post‐injection time points. d) NIR‐II fluorescence imaging of colon and cecum after anatomy. From top to down, control, DSS treatment for 5, 7, 9, 11 days. The signal quantification of colon and cecum and their homogenate signal quantification are subsequently plotted in (e) and (f). The ratio of g) cecum and h) stomach weight to the total mouse was used to further evaluate the severity of colitis in mice. i) H&E‐stained images provided a microanatomy of colon tissue from the control DSS‐treated cohort of mice. The right inset figure illustrates the collected area for microanatomy. All data are expressed as mean ± SD. All data are expressed as mean ± SD.

A particularly important application of our DSPE‐CPDs is to serve as a novel contrast agent for the real‐time assessment of colitis. We thus attempted to evaluate the developmental stages of the colitis model using NIR‐II bioimaging of DSPE‐CPDs. The statistical graph of the abdominal NIR‐II signals can provide a more precise and visualized indicator for grading colitis compared with the DAI score method (**Figure** **6**a). By establishing four cohorts of mice with colitis after DSS treatment for 5, 7, 9, and 11 days, we intragastrically administered DSPE‐CPDs and collected NIR‐II imaging in the supine position regularly over 24 h (Figure 6b). All the statistical NIR‐II signals of the abdomen were plotted in the form of a heat map (Figure 6c), and the results were found to be consistent with DAI prediction, which suggests that cohorts from DSS‐treated for seven days had the highest degree of colitis. The ex vivo dissected cecum and colon after full rinsing with PBS at 12 h post‐injection displayed a result consistent with the in vivo prediction (Figure 6d). In addition, the mean brightness signal of the in vivo abdominal ROI and dissected intestine homogenate showed a close correlation with the DAI score (Figure 6e,f). To further confirm the reliability of the DSPE‐CPDs‐derived colitis indicator for disease severity and inflammatory degree assessment, the cecum and stomach in proportion to the bodyweight of mice were also assessed (Figure 6g,h). All colitis organs from the control and four cohorts of colitis mice were confirmed through H&E staining; as expected, inflammatory lesions were found in the colitis groups (Figure 6i).

We also carried out real‐time disease assessment using DSPE‐R1‐a1 in lipopolysaccharide (LPS)‐induced acute stress inflammatory model through both intragastric and intravenous administration (Figure S13a,b, Supporting Information). NIR‐II imaging results showed that DSPE‐CPDs also enabled high‐quality visualization of the stomach and colon in mice with LPS‐induced inflammation (Figure S13c, Supporting Information). Collectively, DSPE‐CPDs with inherent inflammation imaging ability have the potential for real‐time assessment of the severity of multiple inflammatory diseases.

## 3 Conclusion

The commonly accepted emission mechanism for CPDs is the molecular state, which is derived from the connected luminophore groups on the CPDs.^[^ ^23^ ^]^ Inspired by this mechanism, we designed and manufactured series of NIR‐CPDs through a two‐step hydrothermal/Knoevenagel reaction with adjustable absorption/emission wavelength for NIR‐II imaging. The CPDs’ emission centers were formed by covalently binding the indole derivatives with the crosslinked conjugated carbon core. The emission tails of the prepared NIR‐CPDs can reach up to 1400 nm. The proposed synthesis procedure for NIR‐CPDs is simple and fast, with low‐cost reaction precursors. Compared with cyanine dyes with similar emission centers, the NIR‐CPDs displayed substantially enhanced photostability. Our work also provides an entirely new synthesis strategy for the preclinical development of bright NIR‐II emissive fluorophores, given that the synthesis protocols involved in producing NIR‐II dyes are generally complicated and labor‐intensive. The emission wavelengths of the majority of reported CPDs have been limited in the range of visible to NIR‐I (400−900 nm) region.^[^ ^17^, ^23^ ^]^ In vivo fluorescence imaging in the NIR‐II window can further improve the imaging contrast and penetration depth.

Although our NIR‐CPDs are water‐soluble and can be applied in vivo with confirmed good biocompatibility, both albumin and amphiphilic polymers can considerably improve the NIR‐II brightness of NIR‐CPDs by forming either encapsulated micelles or cocktails. We applied these micelles/cocktails for NIR‐II angiography and bioimaging of gastrointestinal inflammation through a non‐invasive, real‐time, and spatiotemporal route. The encapsulated DSPE‐CPDs preferentially accumulated in the inflammatory microenvironment, possibly due to the enhanced permeability and retention (EPR) effect,^[^ ^24^ ^]^ thus, we achieved a non‐invasive real‐time assessment of disease severity in two types of gastrointestinal inflammation models. The accuracy rate may surpass the standard H&E and DAI score methods. In addition, our method can be administered intravenously or orally. In light of the above, we expect it to have a very important clinical impact.

## Conflict of Interest

The authors declare no conflict of interest.

## Supporting information

Supporting Information

## Data Availability

The data that support the findings of this study are available from the corresponding author upon reasonable request.
