# Supplementary material for: Near‐Infrared Carbonized Polymer Dots for NIR‐II Bioimaging
Source: Adv Sci (Weinh). 2022 Sep 1;9(30):2203474. doi: 10.1002/advs.202203474 (PMC9596834; doi:10.1002/advs.202203474)
Supplement: Supplementary file 1 — Supporting Information [file ADVS-9-2203474-s001.pdf]

ADVANCED SCIENCE
Open Access

Supporting Information

for *Adv. Sci.*, DOI 10.1002/advs.202203474

Near-Infrared Carbonized Polymer Dots for NIR-II Bioimaging

Tianyang Han, Yajun Wang, Shengjie Ma, Mengfei Li, Ningning Zhu, Songyuan Tao, Jiajun Xu, Bin Sun, Yunlong Jia, Yuewei Zhang, Shoujun Zhu and Bai Yang*

Supporting Information

Near-infrared carbonized polymer dots for NIR-II bioimaging

Tianyang Han, Yajun Wang, Shengjie Ma, Mengfei Li, Ningning Zhu, Songyuan Tao, Jiajun Xu, Bin Sun, Yunlong Jia, Yuewei Zhang, Shoujun Zhu, Bai Yang*

Table of Contents

1. Materials and methods
2. Supplementary figures
3. Supplementary tables
4. Synthesis details of indole derivatives
5. NMR spectra of indole derivatives
6. References

1. Materials and methods.

1.1. Preparation of CHO-CPDs and NIR-CPDs.

The CHO-CPDs were prepared based on a modified method sourced from a previous report.[1] 1.0 g aldehyde precursor was dissolved in 8 mL 50% ethanol-water solution and reacted in a hydrothermal reactor at 200°C for 24 h. After cooling to room temperature, the product was washed using a dialysis bag (500 Da) in 1.0 L deionized (DI) water for 48 h (water changed five times per day) or 1.0 L ethanol for 24 h. CPDs-CHO was finally concentrated using an evaporator to obtain a brown cream product and further dispersed in ethanol as stocker solution (200 mg·mL^{-1}).

A portion of the 1.0 g R1-CHO was dissolved in acetic anhydride (10.0 mL), followed by the addition of methyl indole (5.0 g) and sodium acetate (50 mg). After reacting at room temperature for 48 h or 50°C for 8 h, the mixture was poured into DI water. After washing using a dialysis bag (1000 Da) against 1.0 L DI water for 48 h (water changed 5 times per day), the NIR-CPDs (two-step) were collected by freeze-drying or extraction with ethyl acetate (for b-serials only) and brine.

A portion of the 4 mL glutaraldehyde (25%), sodium acetate (50 mg), and 5.0 g 4-(2,3,3-trimethyl-3H-benzo[g]indol-1-ium-1-yl) butane-1-sulfonate were dissolved in acetic anhydride (10.0 mL) and reacted in a hydrothermal reactor at 200°C for 24 h. After cooling to room temperature, the product was washed using a dialysis bag (500 Da) in 1.0 L deionized (DI) water for 48 h (water changed five times per day). The NIR-CPDs (one-step) were collected by freeze-drying.

1.2. The amount of aldehyde was determined using hydroxylamine hydrochloride.

The amount of aldehyde was determined using hydroxylamine hydrochloride titration. Different volumes of R1-CHO stocker were diluted with 25 mL hydroxylamine hydrochloride methyl solution (0.25 mol/L), and corresponding volumes of DI water were added to increase the system volume to 26 mL. The mixture was stirred at room temperature for 1 h to completely dissolve it in a homogeneous solution. An aqueous solution of NaOH with an accurate concentration of 0.1 mol/L was used as the titration reagent. The concentration of the aldehyde group was calculated according to the amount of NaOH.

In this experiment, the concentration values of CPDs-CHO were 0, 0.1, 0.2, 0.5, 1.0, and 2.0 mg·mL^{-1}. Each group of CPDs was titrated with the volume of NaOH as the Y-axis and the concentration of the substance measured as the X-axis until the solution turned yellow (pH = 5.0). After linear fitting, the aldehyde group concentration of CPDs-CHO was calculated using the following equation:

$$C_s(CHO) = \frac{0.1 \times [V(NaOH) - V_0(NaOH)]}{V_S} = k$$

Here, Cs is the concentration of CPDs in the stocker, V(NaOH) is the volume of NaOH (0.1 mmol·mL^{-1}), V_s is the volume of the CPDs-CHO stocker mixed in the system, and k is the slope of the linear fitting.

1.3. Quantum yield test.

The QYs of the fluorophores were measured using previously reported procedures with modifications.[2] Due to batch-to-batch differences in HiPco single-walled carbon nanotubes (SWCNTs) and production processes, the QY of our freshly made HiPco SWCNTs was determined to be between 0.03% to 0.31% with IR-26 (0.05 to 0.5%) as reference (**Figure S4**). The NIR-II fluorescence emission intensities were measured under the same 808 nm excitation. The QY values of the samples were determined on the basis of five concentrations with gradient ODs at 808 nm. Using the measured ODs at 808 nm and the integrated fluorescence intensity, the quantum yield of a test sample was calculated according to the following equation:[2]

$$\varphi_x(\gamma) = \varphi_{std}(\gamma) \times \frac{F_X}{F_{std}} \times \frac{A_{std}(\gamma)}{A_x(\gamma)} \times \left(\frac{\eta_x}{\eta_{std}} \right)^2$$

1.4. Photostability.

NIR-CPDs were dissolved in PBS, FBS, or BSA (50 mg·mL^{-1}). The fluorescence signal was monitored using a two-dimensional InGaAs camera under continuous exposure to an 808-nm laser at a power density of 63.2 mW/cm^2. The average fluorescence intensity of the region of interest (ROI) was plotted as a function of time.

1.5. Cell viability assessment using the 3-(4,5-Dimethylthiazol-2-yl)-2,5-diphenyltetrazolium bromide (MTT) colorimetric assay.

The 4T1, U87, HeLa, and L-02 cells (5k per well) were seeded into a 96-well plate (NEST) and incubated for 12 h at 37 °C in a humidified incubator with 5% CO_2. Then, DMEM solutions with 0.01, 0.02, 0.05, 0.1, and 0.5 mg·mL^{-1} NIR-CPDs and DSPE-R1-a1 were added. After 24 h of incubation, the cells were rinsed three times with PBS and 100 µL MTT (0.5 mg·mL^{-1}) solution was added. After removing the MTT solution after 4 h of incubation, 50 µL DMSO was added to each well. Placing the shaking table at a low speed for 2 h allowed the crystal to fully dissolve. The absorbance value of each well was measured at OD 570 nm using an Elisa reader (BioTek Synergy LX).

1.6. Animals and ulcerative colitis models.

All animal experiments were conducted under institutional guidelines and were approved by the Experimental Animal Ethical Committee of the First Hospital of Jilin University (Protocol number: 20210642). BALB/c mice were purchased from Liaoning Changsheng Biotechnology Co. Ltd. Bedding, nesting materials, food, and water were provided ad libitum. The ambient temperature was controlled between 20 °C to 24 °C. To attenuate the interference of NIR fluorescent substances in mouse feed on gastrointestinal imaging, all mice used for *in vivo* imaging were given jelly for food 24 h before the experiment.

For ulcerative colitis models, 30.0 g DSS was dissolved in 1 L DI water to prepare a 3% DSS solution. Mice were randomly divided into control group (n = 6) and DSS group (n = 6). The body weight and excretion of the mice were observed and recorded. The drinking water of the experimental group was replaced by the 3% DSS

group on day 0 of the modeling. The drinking water was replaced with DI water on day 7.[3] The weight and excretion of the mice were continuously observed and recorded over the next 14 days. The disease activity index (DAI) score was used to evaluate the degree of inflammation in the model mice cohort. The DAI scores were calculated according to previous reports.[3] Briefly, DAI scores were calculated based on the average score of body weight loss, feces consistency, and fecal blood test scores (**Table S2**).

1.7. Experiment with colitis models.

Mice were sacrificed 12 h after administration, in addition to those used to observe long-term imaging (n = 3 in for each group). The entire colon was quickly removed from the cecum to the anus. Colons from the imaging group at the desired time points were collected and randomly assigned to three groups. In the first group, the portion was divided into the colon and cecum, and both were weighed, washed, and imaged using a NIR camera. Furthermore, the tissues were broken up into tissue homogenates using liquid nitrogen freezing and ultrasonic machines. The homogenates were placed in small centrifuge tubes to measure the NIR-II signal. In the second group, the colon was not washed and was directly subjected to NIR imaging or preparation of homogenates. Organs from the third group were fixed in 4% paraformaldehyde for histopathological examination.[3a]

1.8. H&E staining.

All the tissues were fixed in 4% paraformaldehyde after harvesting. These tissues were further dehydrated, embedded in paraffin, and sectioned into 3 μm thick slides. H&E staining was then performed according to the protocol of the H&E kit (Beyotime Institute of Biotechnology, Cat. No. C0105). H&E staining images of every tissue were acquired using the Nikon Eclipse 80i microscope.

1.9. NIR-II imaging.

All mice were shaved using Nair depilatory cream and anesthetized with chloral hydrate or isoflurane before the experiment, and then placed on the imaging table. At least three mice were used as parallel controls in each imaging experiment. All NIR-I/NIR-II images were collected on a two-dimensional InGaAs array (Princeton Instruments, NIRvana-640) with a laser wavelength of 808 nm and a power density of 63.2 mW/cm^2.

1.10. Metabolism assessment.

Under 808 nm excitation, 900 and 1000 nm long-pass filters were used to collect NIR-II imaging under the InGaAs camera. NIR-CPDs suspended in PBS (0.1 $mg \cdot mL^{-1}$, 200 μL) were intravenously injected into six-week-old BALB/c mice (n = 3). Images were obtained at 0 min, 30 min, 1 h, 3 h, 6 h, 12 h, 24 h, and 48 h post-injection. For DSPE-R1-a1, the injection of cocktail included 0.1 $mg \cdot mL^{-1}$ CPDs and 0.5 $mg \cdot mL^{-1}$ DSPE-mPEG$_{2000}$ (200 μL).

1.11. High-performance NIR-II angiography.

Under 808 nm excitation, different long-pass filters (1000, 1100, and 1200 nm) were used to collect the NIR-II imaging under the InGaAs camera. The combination of an 850 nm long-pass filter and a 1000 nm short-pass filter was used to collect the NIR-I imaging. NIR-CPDs suspended in PBS (3.0 $mg \cdot mL^{-1}$, 200 μL) were intravenously injected into six-week-old C57 mice (n = 3). Video-rate imaging was performed immediately after the injection to monitor the blood perfusion in real-time for the first 15 min.

1.12. NIR-II imaging of acute colitis.

Under 808 nm excitation, 900 and 1000 nm long-pass filters were used to collect the NIR-II imaging under the InGaAs camera. DSPE-R1-a1 suspended in PBS (0.1 $mg \cdot mL^{-1}$, 200 μL) was intravenously or intragastrically injected into six-week-old BALB/c mice (n = 3). The NIR-II images were collected at designated post-injection time points.

1.13. Statistical Analysis.

All data are expressed as mean ± SD (n = 3 or 6 for biological experiment signal acquisition; n = 6 for optical in vitro characterization). For normally distributed data sets with equal variances, one-way ANOVA testing followed by a Tukey post-hoc test was carried out across groups. In all cases, significance was defined as $^{*}p < 0.05$, $^{**}p < 0.01$, $^{***}p < 0.001$. The luminescence intensity of the dye in the photostability experiment was normalized according to the intensity at 0 min. The diagram of the absorption and emission spectrum is normalized according to the intensity of the absorption spectrum. Statistical analysis was carried out using OriginPro 2018 and Excel 2021.

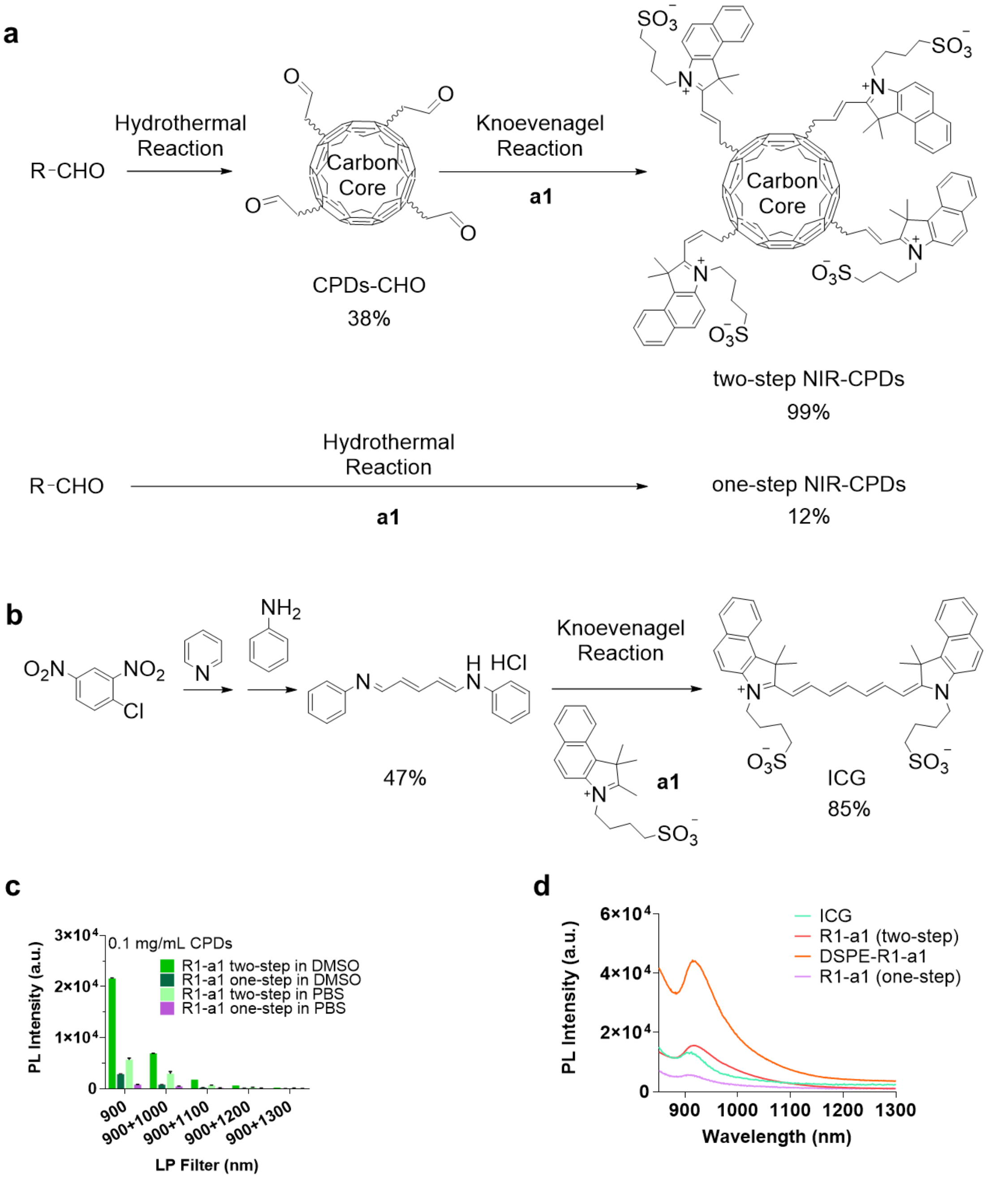

e

	Commercial price (g)	Synthesize cost (g)	Purification method
ICG	5600.00 ¥	752.00 ¥	Silica gel chromatography
R1-a1	-	160.00 ¥	dialysis

2. Supplementary figures

Figure S1. NIR-CPDs show a much lower cost and easier synthesis procedure

compared with commercial NIR dye with the same a1 side chain. a) Production yield of NIR-CPDs through one-step and two-step methods, and commercial b) ICG fluorophore. c) NIR-II brightness of CPDs synthesized via one or two steps in the DMSO/PBS buffer under different long-pass filters (900−1300 nm). d) NIR-CPDs micelles showed higher NIR fluorescence than ICG. e) The synthesis cost is approximately calculated based on the raw material prices from the TCI company (the yield of ICG has been obtained from the literature).[4] Data are mean ± SD.

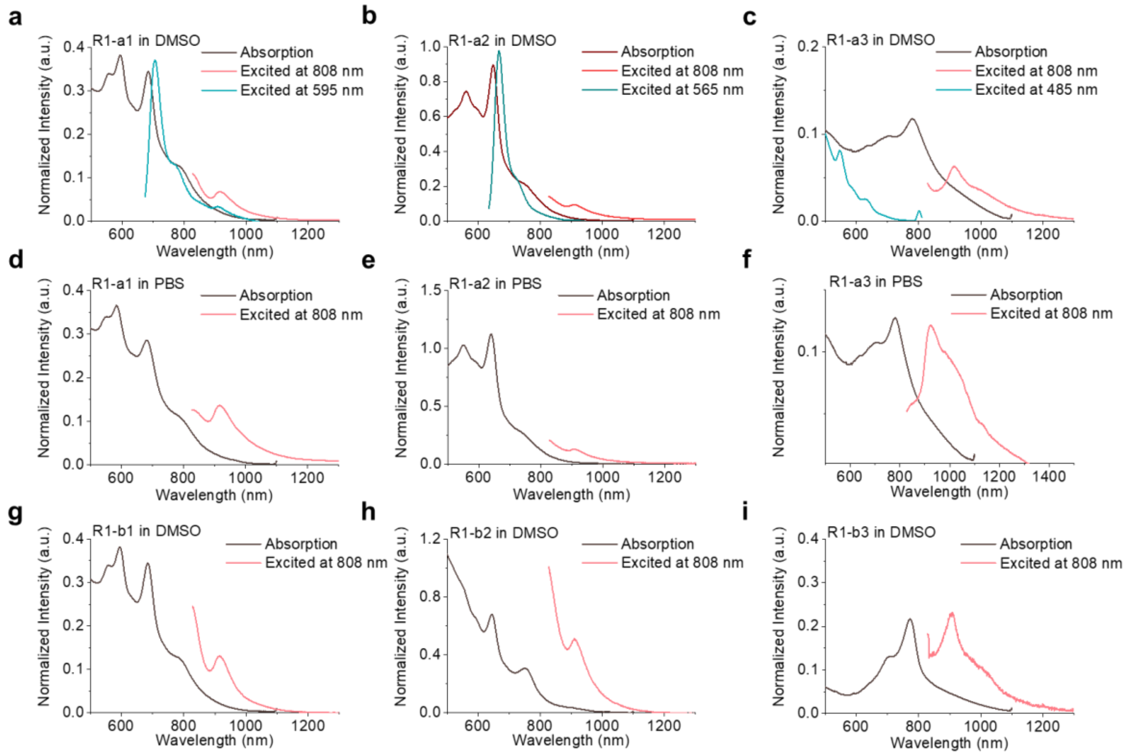

Figure S2. Absorption and emission spectra of NIR-CPDs (R1-a1, R1-a2, R1-a3, R1-b1, R1-b2, and R1-b3). The a-serial NIR-CPDs, which are water-soluble, were recorded in both a) b) c) DMSO and d) e) f) PBS buffer. However, b-serial NIR-CPDs, which are insoluble in water, were tested in g) h) i) DMSO only. The emission spectrum is normalized according to the intensity of the absorption spectrum.

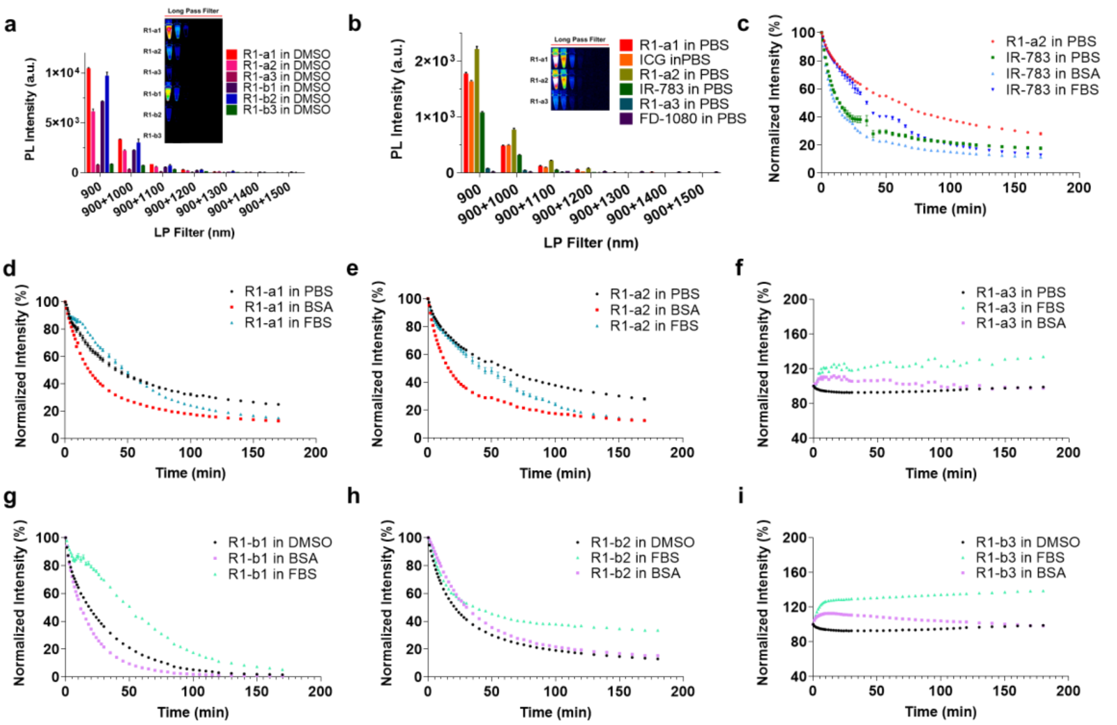

Figure S3. Optical properties of NIR-CPDs. a) Fluorescence intensity of NIR-CPDs in DMSO under different sub-NIR-II windows (normalized at OD 808 nm = 0.1). Insets are fluorescence imaging pictures of NIR-CPDs in DMSO under different sub-NIR-II windows. b) Fluorescence intensity comparison between NIR-CPDs and commercial NIR dyes in PBS (normalized at OD 808 nm = 0.1). Insets are fluorescence imaging pictures of NIR-CPDs in PBS under different sub-NIR-II windows. c) Photostability of NIR-CPDs R1-a2 in PBS and commercial NIR dye with equivalent OD at 808 nm in PBS, BSA (50 mg·mL^{-1}), and FBS. d-f) Photostability of 0.1 mg·mL^{-1} a-serials NIR-CPDs in PBS, BSA (50 mg·mL^{-1}), and FBS, respectively. g-i) Photostability of 0.1 mg·mL^{-1} b-serials NIR-CPDs in PBS, BSA (50 mg·mL^{-1}), and FBS, respectively. Imaging condition for photostability: 808 nm laser excitation with 63.2 mW/cm^2 power density, 900 nm long-pass filters. Continuous variables are expressed as mean ± SD. The luminescence intensity of the dye in the photostability experiment was normalized according to the intensity at 0 min.

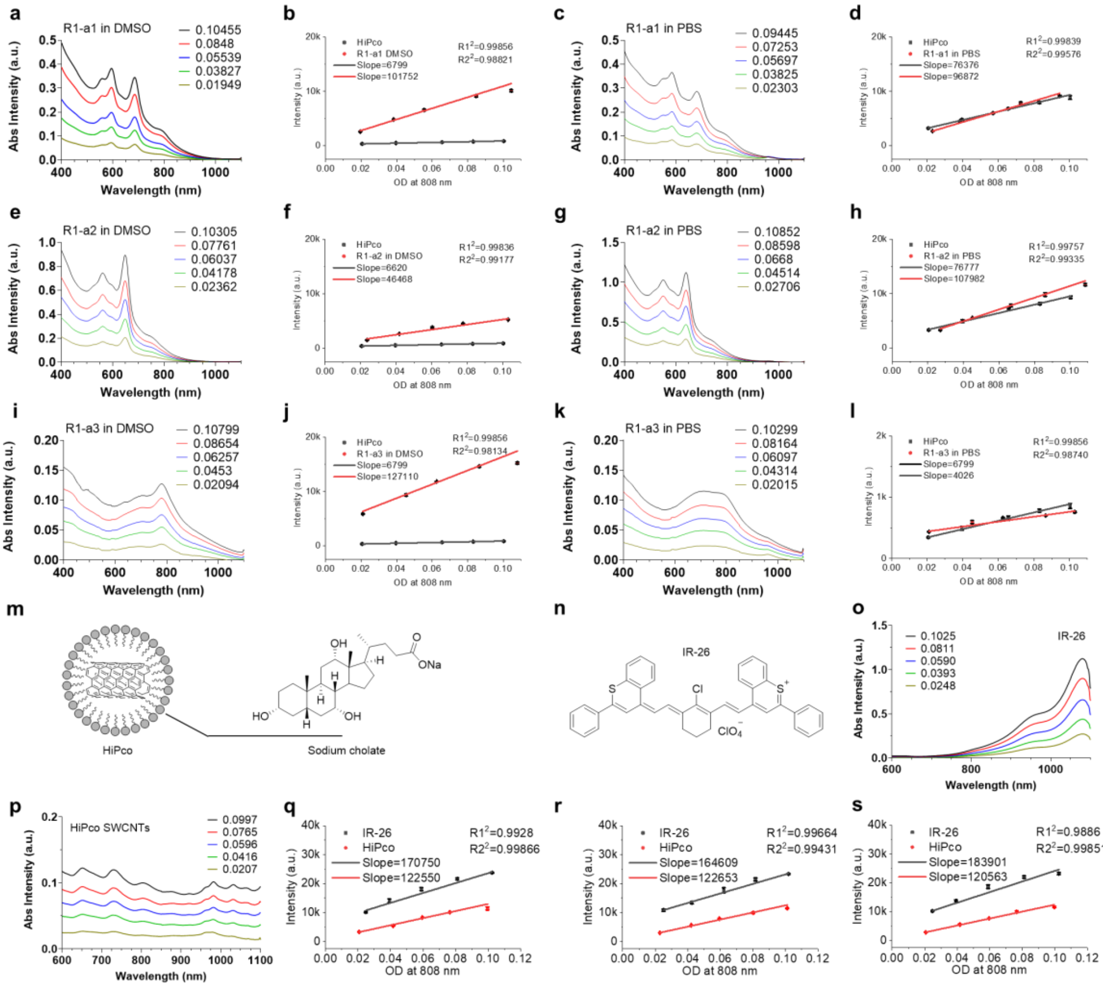

Figure S4. NIR-II quantum yields (QYs) of CPDs (R1-a1, R1-a2, and R1-a3) were determined using HiPco SWCNTs as the reference. Optical absorbance spectra of a series of five solutions of CPDs in either DMSO (a, e, i) or PBS (c, g, k) with absorbance values at 808 nm were in the range from ~0.02 to ~0.1. The integrated NIR-II signal intensities (900−1500 nm) of the above samples plotted against the absorbance at 808 nm for R1-a1 in b) DMSO and d) PBS, R1-a2 in f) DMSO and h) PBS, R1-a3 in j) DMSO and l) PBS, respectively. The QYs of the freshly made m) HiPco SWCNTs were first corrected by n) IR-26 dye in chloroform and DCE. The QY of IR-26 is not consistent in the literature; thus, we chose a range from 0.05% to 0.5% in this study.[6] o-s) Due to batch-to-batch differences in original SWCNTs and production processes, the QY of our freshly made HiPco SWCNTs was determined to be 0.031% to 0.31% with IR-26 (0.05% to 0.5%). HiPco SWCNTs were dissolved in sodium cholate solution (10 mg·mL^{-1}) and IR-26 was dissolved in 1,2-dichloroethane (DCE). All data are expressed as mean ± SD. For normally distributed data sets with equal variances, one-way ANOVA testing followed by a Tukey post-hoc test was carried out across groups.

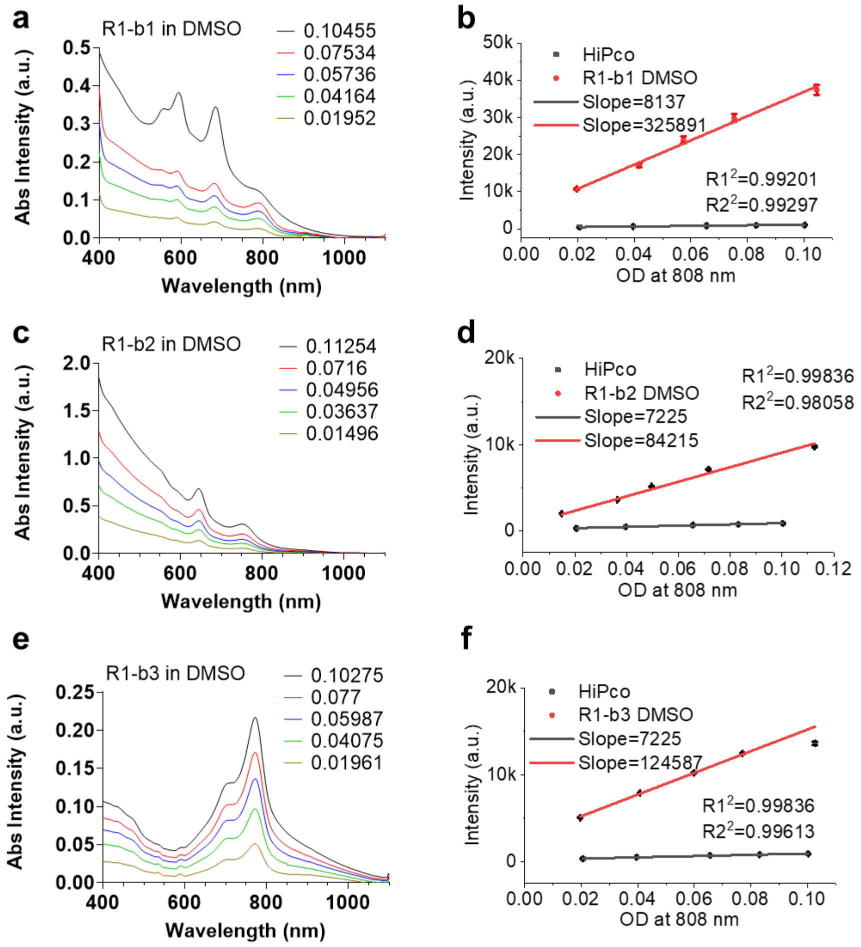

Figure S5. NIR-II quantum yields (QYs) of CPDs (R1-b1, R1-b2, and R1-b3) were determined using HiPco SWCNTs as the reference. Absorption spectra of five concentrations of a) R1-b1 c) R1-b2 e) R1-b3 in DMSO. The integrated NIR-II intensities (900−1500 nm) of the above samples plotted against the absorbance at 808 nm for b) R1-b1 d) R1-b2 f) R1-b3 in DMSO. All data are expressed as mean ± SD. For normally distributed data sets with equal variances, one-way ANOVA testing followed by a Tukey post-hoc test was carried out across groups.

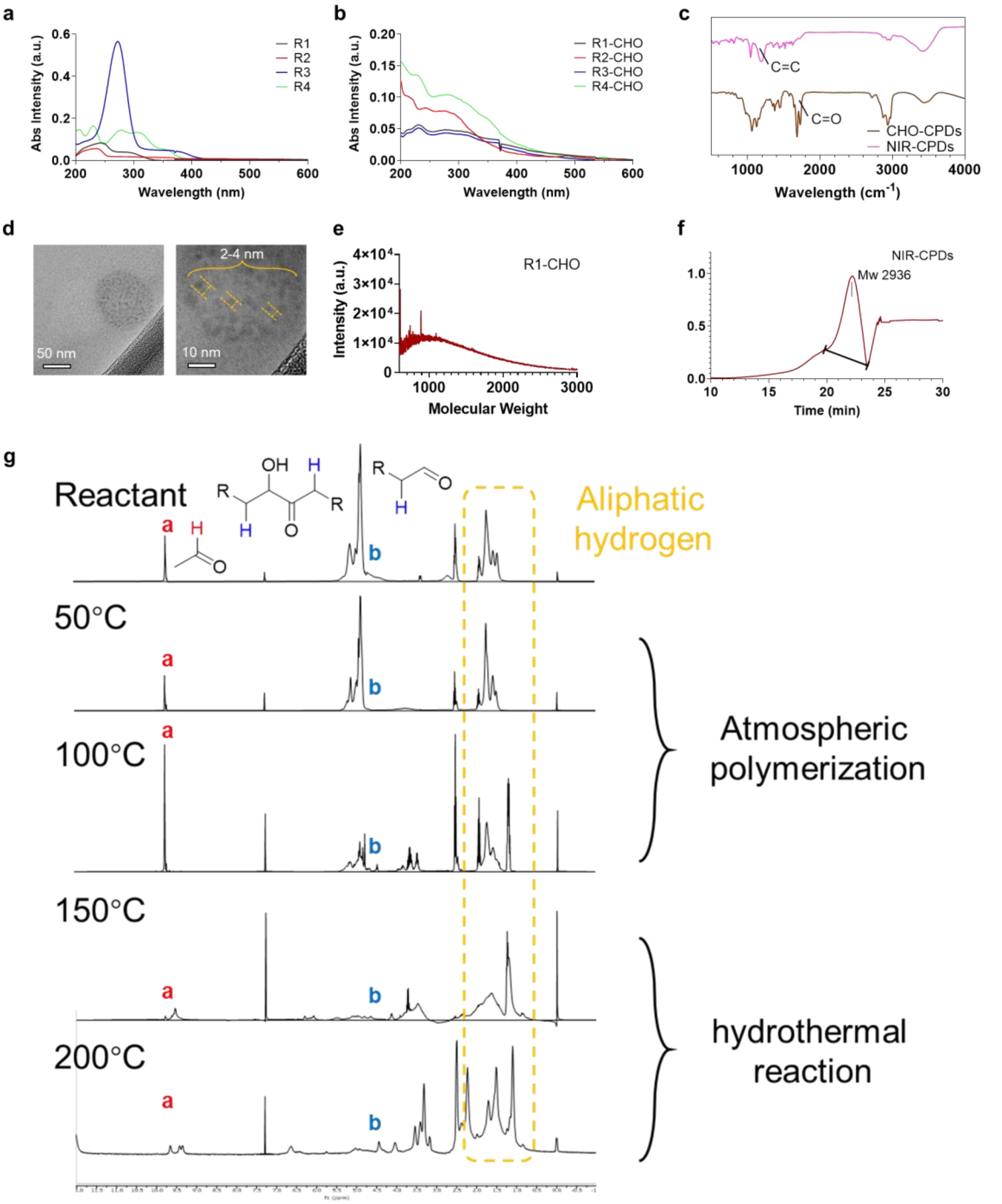

Figure S6. Other chemical structure characterization of CPDs-CHO and NIR-CPDs. UV-Vis absorption spectrum of 0.1 $mg \cdot mL^{-1}$ a) carbon precursors of CPDs-CHO and b) CPDs-CHO in EtOH. c) Fourier transform infrared spectroscopy (FTIR) of CPDs-CHO and NIR-CPDs. d) Transmission electron microscope (TEM) image of CPDs R1-a1. The ZETA potential of -10 mV was obtained. The average molecular weight of CPDs-CHO and NIR-CPDs were checked using e) MALDI-TOF and f) GPC, respectively. g) ^{1}H NMR of glutaraldehyde reacted under different conditions.

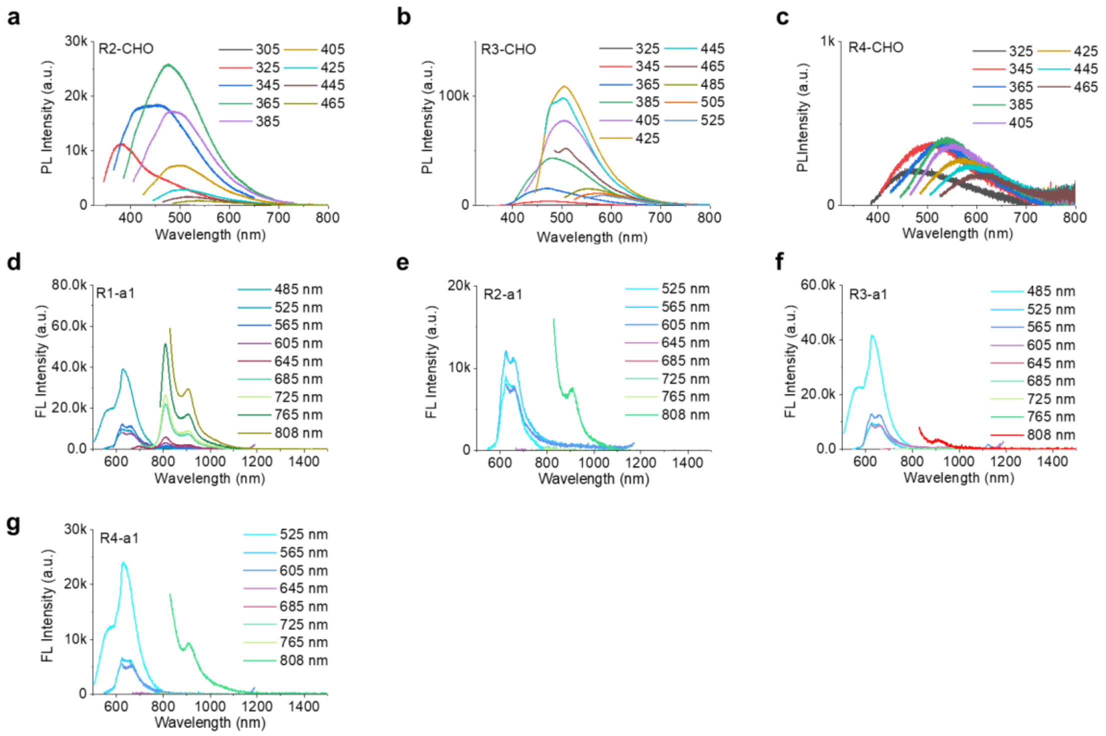

Figure S7. Photoluminescence emission spectra of CPDs-CHO and NIR-CPDs. Photoluminescence emission spectra of CPDs-CHO: a) R2-CHO, b) R3-CHO, and c) R4-CHO. The fluorescence emission spectra of NIR-CPDs: d) R1-a1, e) R2-a1, f) R3-a1, and g) R4-a1.

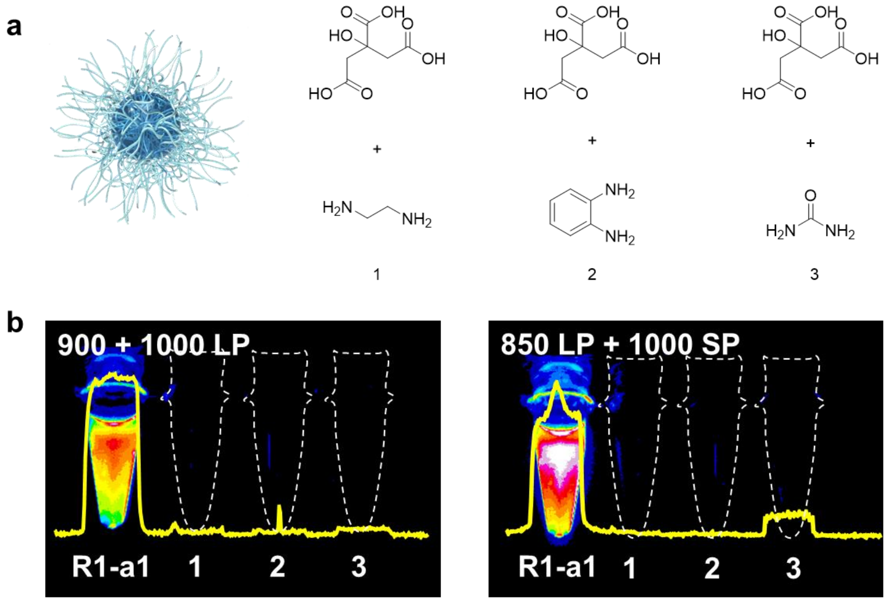

Figure S8. NIR-II brightness of NIR-CPDs R1-a1 compared with previously reported CPDs. From left to right, NIR-CPDs R1-a1, CPDs synthesized using a) citric acid and ethylenediamine, CPDs synthesized using citric acid and phenylenediamine, and CPDs synthesized using citric acid and urea.[7] b) The NIR-II brightness of CPDs was compared through NIR Camera.

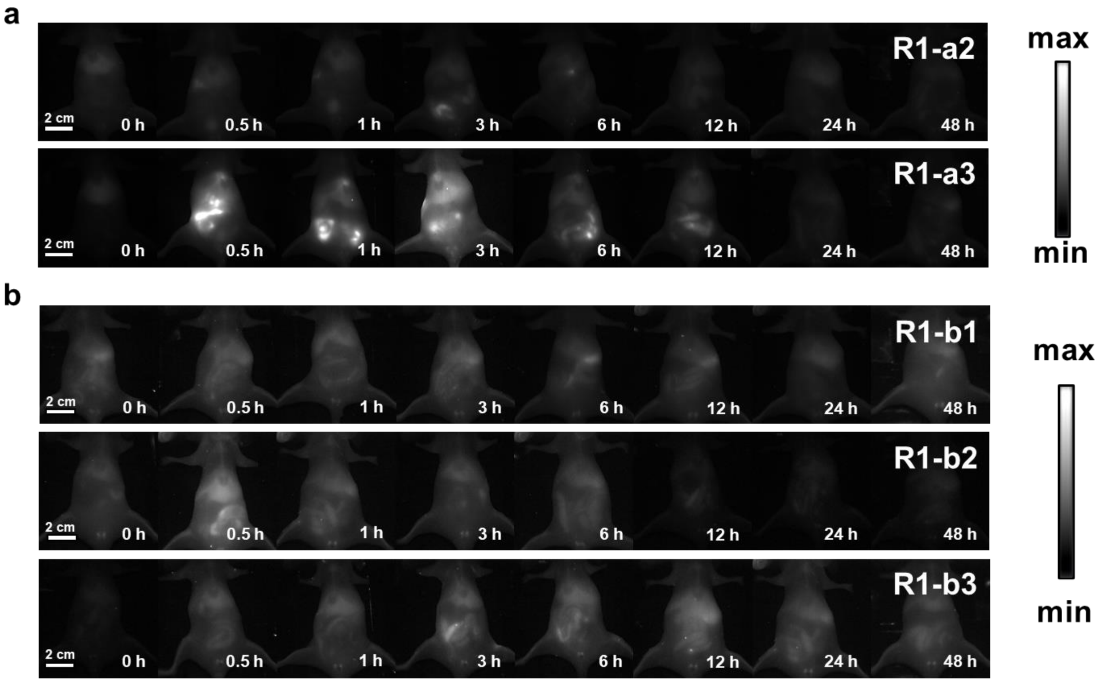

Figure S9. NIR-II whole-body imaging using two sets of NIR-CPDs. NIR-II fluorescence time points (imaging condition: 808 nm laser excitation with 63.2 mW/cm^2 power density, 900 + 1000 nm long-pass filters) of mice in the supine position after intravenous administration of a) a-serial and b) b-serials NIR-CPDs taken as a function of injected time (injection dosage: 0.1 $mg\cdot mL^{-1}$, 200 μL).

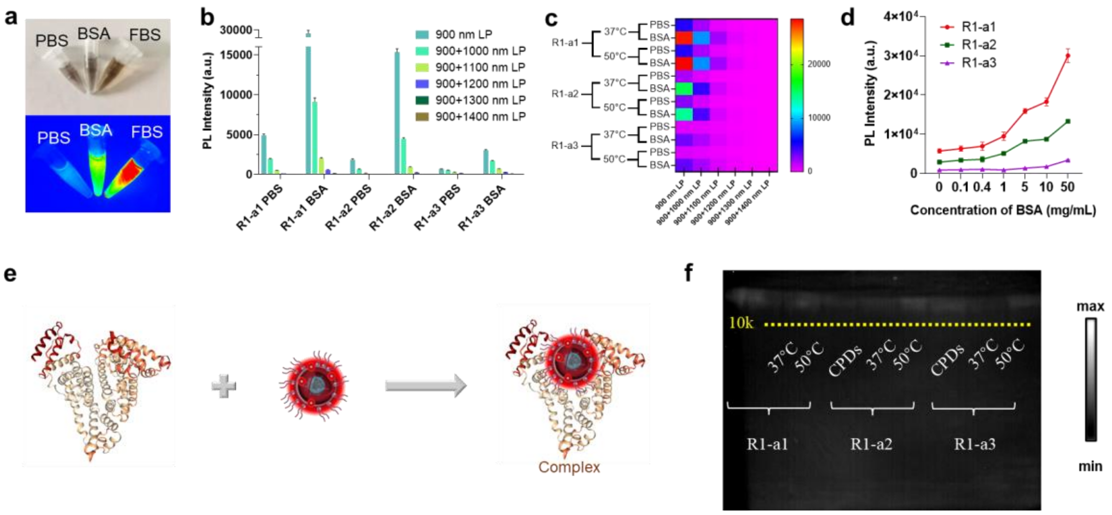

Figure S10. Complexation between NIR-CPDs and albumin enabled high-performance NIR-II angiography. a) NIR-CPDs show enhancement in NIR-II signal when mixed with either BSA (50 mg·mL^{-1}) or FBS. b-c) Fluorescence intensity of a set of NIR-CPDs in PBS and BSA (50 mg·mL^{-1}) solutions under relevant sub-NIR-II windows. d) Fluorescence intensity of NIR-CPDs in different concentrations of BSA solution after incubation at 37°C or 50°C for 4 h. e) Sketch picture of protein complexation of NIR-CPDs. f) Protein electrophoresis of free NIR-CPDs and their mixture with BSA after heating indicated that there was no covalent binding between NIR-CPDs and BSA. Imaging condition: 808 nm laser excitation with 63.2 mW/cm^2 power density. All data are expressed as mean ± SD.

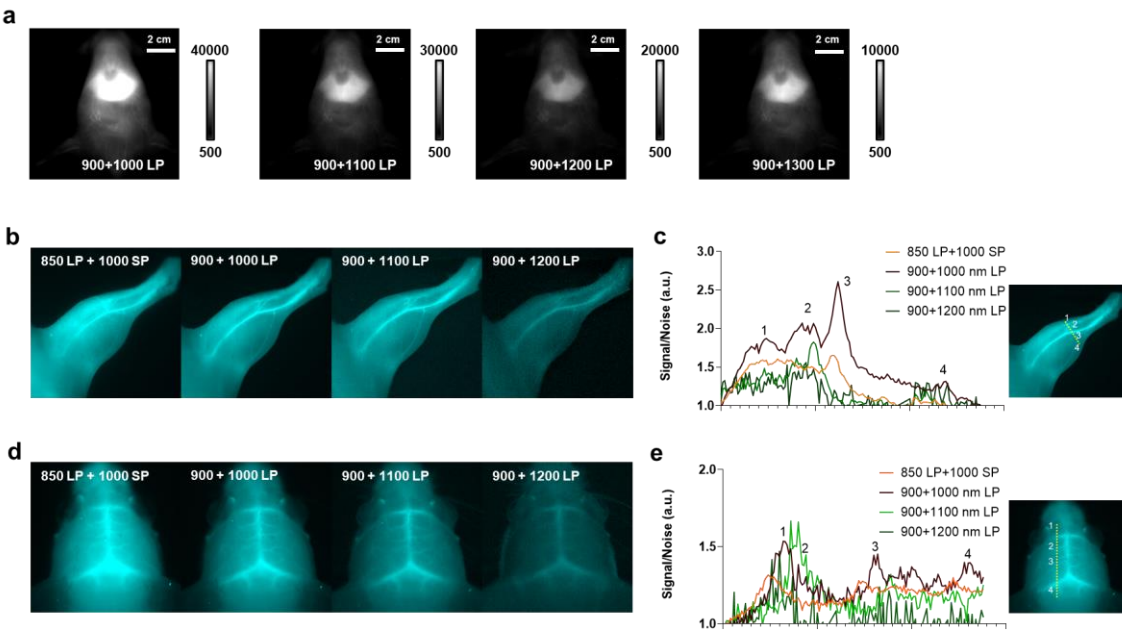

Figure S11. NIR-CPDs enabled high-performance NIR-II angiography. a) NIR-II fluorescence (900 + 1000, 900 + 1100, 900 + 1200, and 900 + 1300 nm long-pass filters) of mice in the supine position after intravenous administration of NIR-CPDs R1-a1 (injection dosage: 3 mg·mL^{-1}, 200 μL). b) NIR-II imaging of a mouse hindlimb after intravenous injection of NIR-CPDs R1-a1 under the NIR-I window and different NIR-II (1000, 1100, and 1200 nm) windows. c) The signal-to-noise (S/N) ratio was quantified by plotting the amplitude of the hindlimb after extracting the background signal from perivascular skin. d) NIR-II imaging of mouse cerebrovascular after intravenous injection with NIR-CPDs R1-a1 under both NIR-I window (850-1000 nm) and sub-NIR-II (>1000, >1100, and >1200 nm) windows. e) S/N ratio of cerebrovascular was calculated by plotting the cross-sectional profile after extracting the background signal. Imaging condition: 808 nm laser excitation with 63.2 mW/cm^2 power density; exposure time was 5 ms for (a) and 20 ms for (b-e).

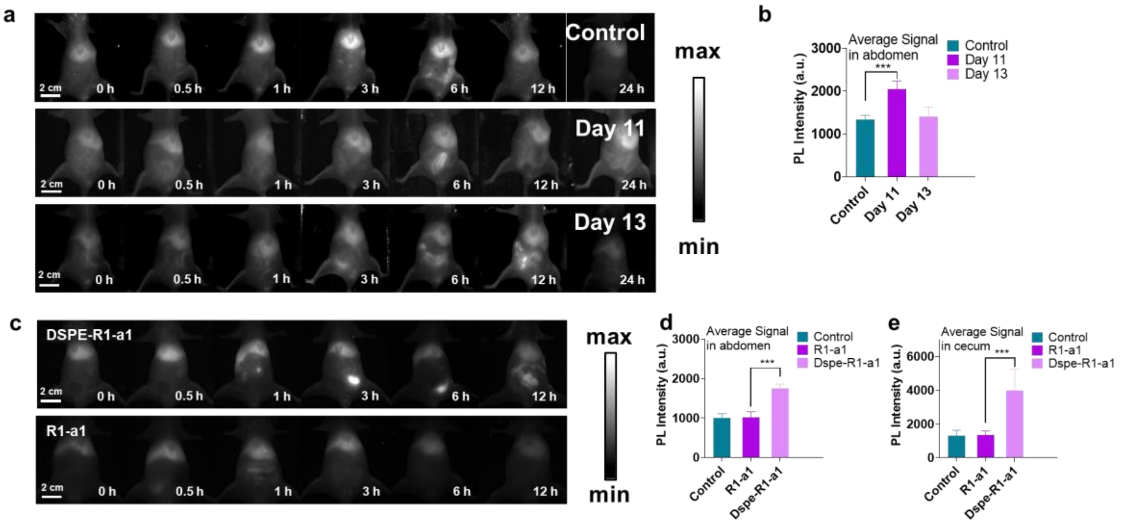

Figure S12. a) NIR-II fluorescence time points of colitis mice and control group in the supine position after intravenous administration of DSPE-R1-a1. b) Average NIR-II fluorescence intensity at 12 h post-injection time point of the abdomen from two selected DSS-treated cohorts of mice (day 11 and day 13 groups). c) NIR-II fluorescence time points of colitis mice in the supine position after intravenous administration of DSPE-R1-a1 and free R1-a1 (at the same dosage: 0.1 mg·mL^{-1}, 200 μL). The NIR fluorescence intensities were compared through average NIR fluorescence intensity of d) *in vivo* abdomen ROI and e) *ex vivo* cecum excision. Imaging condition: 808 nm laser excitation with 63.2 mW/cm^2 power density, 900 + 1000 nm long-pass filters. Data are mean ± SD. A statistically significant difference between groups was confirmed by a one-way analysis of variance (ANOVA) test (***p < 0.001).

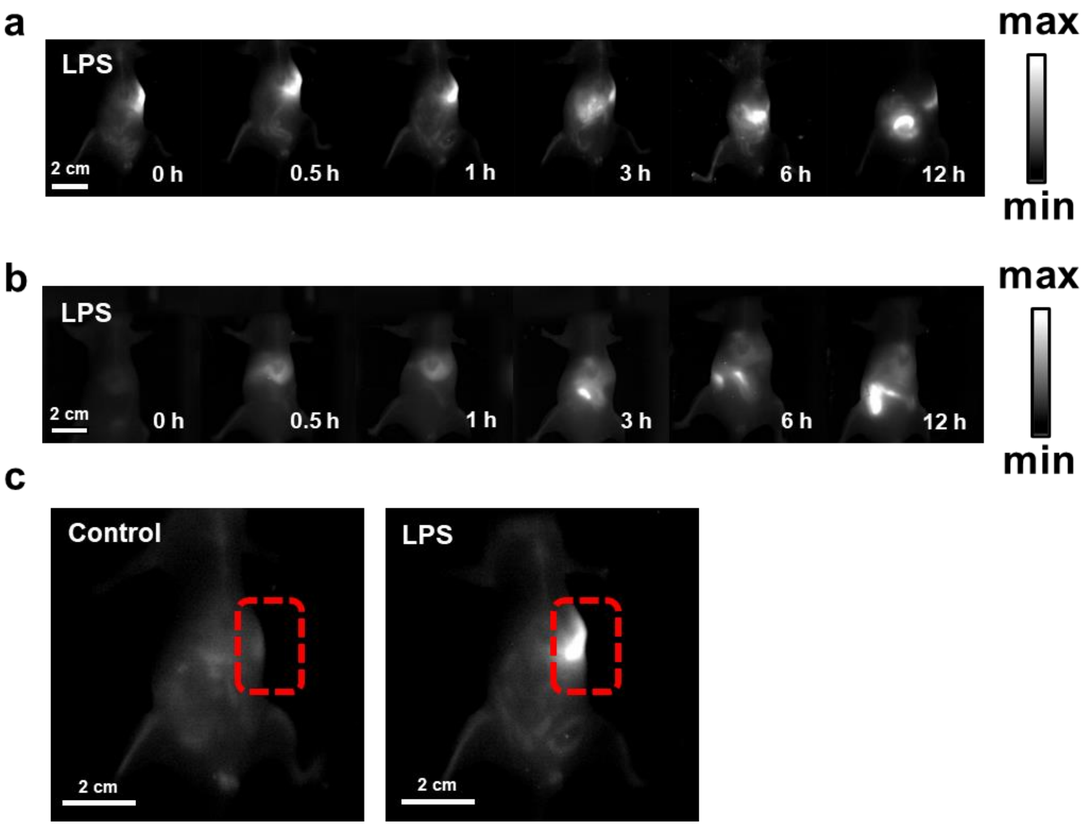

Figure S13. NIR-II imaging of acute colitis (LPS-treated model) using NIR-CPDs. a) NIR-II fluorescence time points of lipopolysaccharide (LPS)-treated mice in the supine position after intragastric administration of DSPE-R1-a1. b) NIR-II fluorescence time points of LPS-treated mice in the supine position after intravenous administration of DSPE-R1-a1. DSPE-R1-a1 showed c) stronger NIR-II signal in the stomach of LPS-treated mice compared with the control group. Imaging condition: 808 nm laser excitation with 63.2 mW/cm^2 power density, 900 + 1000 nm long-pass filters.

3. Supplementary tables

Table S1. Summary of optical properties of NIR-CPDs. (The QYs were tested using HiPco SWCNTs as reference. To eliminate batch deviation, the freshly made HiPco SWCNTs were first corrected using IR-26 (0.05 to 0.5%))

Dye	Solvent	λ (Abs/Em)	QYs (%)	ε ($1 \cdot mg^{-1}$)
R1-a1	DMSO	685 nm/915 nm	0.39 to 3.80[a]	1602
	PBS	685 nm/916 nm	0.04 to 0.40	2061
R1-a2	DMSO	647 nm/ 907 nm	0.18 to 1.80	193
	PBS	639 nm/908 nm	0.04 to 0.39	1866
R1-a3	DMSO	781 nm/913 nm	0.48 to 4.78	5806
	PBS	781 nm/929 nm	0.02 to 0.19	18586
R1-b1	DMSO	685 nm/914 nm	1.03 to 10.25	1374
R1-b2	DMSO	644 nm/911 nm	0.30 to 2.98	805
R1-b3	DMSO	772 nm/908 nm	0.44 to 4.41	16469

The QYs of the freshly made HiPco SWCNTs were first corrected using the IR-26 dye in chloroform and 1,2-dichloroethane (DCE). The QY of IR-26 is not consistent in the literature; thus, we listed a range because QY of IR-26 has variations up to an order of magnitude from 0.05 to 0.5%.[2, 5]

Table S2. Disease activity index (DAI scoring system).

DAI score	Weight loss (%)	Stool consistency	Occult/gross bleeding
0	None	Normal	Normal
1	1−8		
2	5−10	Loose stools	Hemoccult positive
3	10−20		
4	>20	Diarrhea	Gross bleeding

4. Synthesis details of indole derivatives

Synthesis of methyl-indole (Figure S14).

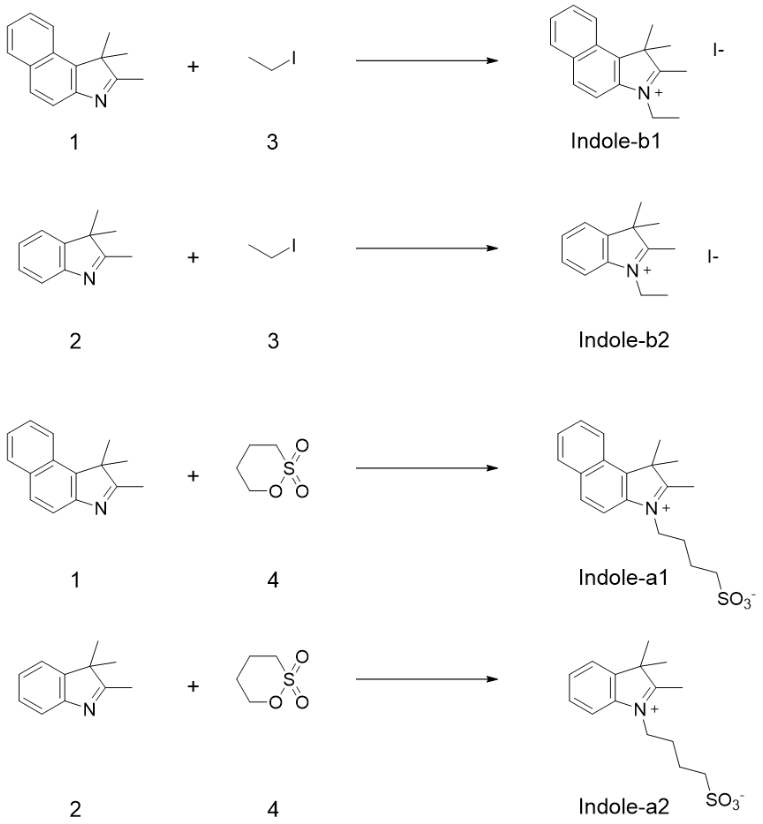

Synthesis of 3-ethyl-1,1,2-trimethyl-1H-benzo[e]indol-3-ium (Indole-b1).

A portion of 1,1,2-Trimethyl-1H-benz[e]indole (1.046 g, 5.0 mmol) was dissolved in acetonitrile (15.0 mL), followed by the addition of iodoethane (1.560 g, 10.0 mmol). With constant stirring, the mixture was refluxed for 24 h. The mixture precipitated naturally in the system and was collected through filtration. After washing with ethyl acetate (EA), the product (84.73 %) was dried in vacuo as a light blue solid. MS: [M^+], m/z = 237.24. ^{1}H NMR (400 MHz, DMSO-d_6) δ 8.40 (d, J = 8.4 Hz, 1H), 8.33 (d, J = 8.9 Hz, 1H), 8.25 (d, J = 8.0 Hz, 1H), 8.18 (d, J = 8.9 Hz, 1H), 7.83-7.73 (m, 2H), 4.64 (q, J = 7.3 Hz, 2H), 1.78 (s, 6H), 1.52 (t, J = 7.3 Hz, 3H).

Synthesis of 3-ethyl-1,1,2-trimethyl-1H-benzo[e]indol-3-ium (Indole-b2).

A portion of 2,3,3-trimethylindolenine (318.46 mg, 2.0 mmol) was dissolved in iodoethane (779.83 mg, 5 mmol). The mixture was allowed to react for 3 h at reflux temperature. The mixture precipitated naturally in the system and was collected through filtration. After washing with EA, the product (70.43 %) was dried in vacuo (pink solid). MS: [M^+], m/z = 187.09. ^{1}H NMR (400 MHz, DMSO-d_6) δ 7.99 (d, J = 8.4 Hz, 1H), 7.86 (d, J = 8.4 Hz, 1H), 7.66-7.64 (m, 2H), 4.51 (q, J = 7.3 Hz, 2H), 1.53 (s, 6H), 1.44 (t, J = 7.3 Hz, 3H).

Synthesis of 4-(2,3,3-trimethyl-3H-benzo[g]indol-1-ium-1-yl) butane-1-sulfonate (Indole-a1).

A portion of 1,1,2-trimethyl-1H-benz[e]indole (1.046 g, 5.0 mmol) was added into a mixture of acetonitrile (5.0 mL) and 1,4-butane sultone (2.72 g, 20 mmol). With

constant stirring, the mixture was refluxed for 72 h. After cooling, the mixture was poured into ether (30 mL). A portion of hexane (5 mL) was added to the system in batches with stirring to obtain a purple precipitate. The product (74.33 %, dark blue) was collected by filtration and crystallized using 20 mL acetone/acetonitrile (20/80). MS: [M^+], m/z = 344.44. ^{1}H NMR (400 MHz, $CDCl_3$-d_6) δ 8.02 (d, J = 8.0 Hz, 1H), 7.95 (d, J = 8.0 Hz,1H), 7.86 (d, J = 8.0 Hz,1H), 7.79 (d, J = 8.0 Hz,1H), 7.55 (m, 1H), 7.45 (m, 1H), 4.55 (m, 2H), 3.16 (m, 2H), 2.41 (s, 2H), 2.25 (m, 2H), 1.87-1.82 (m, 2H), 1.56 (s, 6H) .

Synthesis of 4-(2,3,3-trimethyl-3H-indol-1-ium-1-yl) butane-1-sulfonate (Indole-a2).

A portion of 2,3,3-trimethylindolenine (318.46 mg, 2.0 mmol) was added to a mixture of acetonitrile (0.5 mL) and 1,4-butane sultone (272.34 mg, 2 mmol). With constant stirring, the mixture was refluxed for 72 h. After cooling, the mixture was poured into ether (5 mL). A portion of hexane (2 mL) was added to the reaction system in batches with stirring to obtain a purple precipitate. The product (65.50 %, light purple solid) was collected by filtration and crystallized using 5 mL acetone/acetonitrile (20/80). MS: [M^+], m/z = 294.20. ^{1}H NMR (400 MHz, DMSO-d_6) δ 8.06-8.04 (m, 1H), 7.86-7.83 (m, 1H), 7.65-7.62 (m, 2H), 4.50 (t, J = 8.0 Hz, 2H), 2.00-1.97 (m, 2H), 1.79-1.74 (s, 2H), 1.55 (m, 6H).

Synthesis of 1-ethyl-2-methylbenzo[cd]indol-1-ium (Indole-b3).

1-ethyl-2-methylbenzo[cd]indol-1-ium was synthesized based on a previous protocol[3b]. MS: [M^+], m/z = 196.1. ^{1}H NMR (400 MHz, DMSO-d_6) δ 8.92 (d, J = 8.0 Hz, 1H), 8.78 (d, J = 8.0 Hz,1H), 8.46 (dd, J = 4.0 Hz, 2H), 8.19 (d, J = 8.0 Hz, *1*H), 8.03 (dd, J = 4.0 Hz, 1H), 4.8 (q, J = 8.0 Hz, 2H), 1.71 (t, J = 8 Hz, 3H).

Synthesis of 4-(2-methylbenzo[cd]indol-1-ium-1-yl) butane-1-sulfonate (Indole-a3).

4-(2-methylbenzo[cd]indol-1-ium-1-yl) butane-1-sulfonate was synthesized following a previously reported method.[8] MS: [M^+], m/z = 302.38. ^{1}H NMR (400 MHz, DMSO-d_6) δ 8.35 (d, J = 8.0 Hz, 1H), 8.23 (d, J = 8.0 Hz,1H), 7.95 (dd, J = 4.0 Hz, 2H), 7.70 (t, J = 8.0 Hz, *1*H), 7.62 (t, J = 8.0 Hz, *1*H), 4.44 (q, J = 8.0 Hz, 2H), 3.52 (t, J = 8.0 Hz, 2H), 3.83 (m, 2H), 2.90 (m, 2H).

5. NMR spectra of the indole derivatives

Figure S15 ^{1}H NMR of indole derivatives.

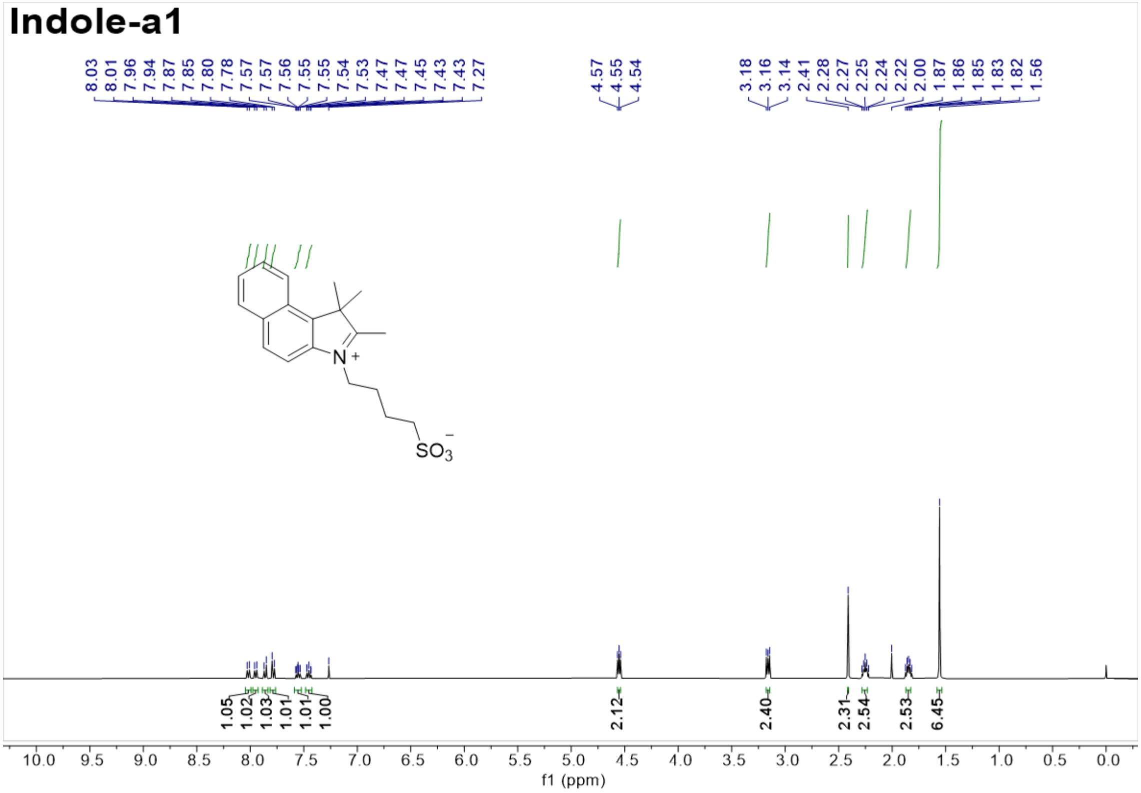

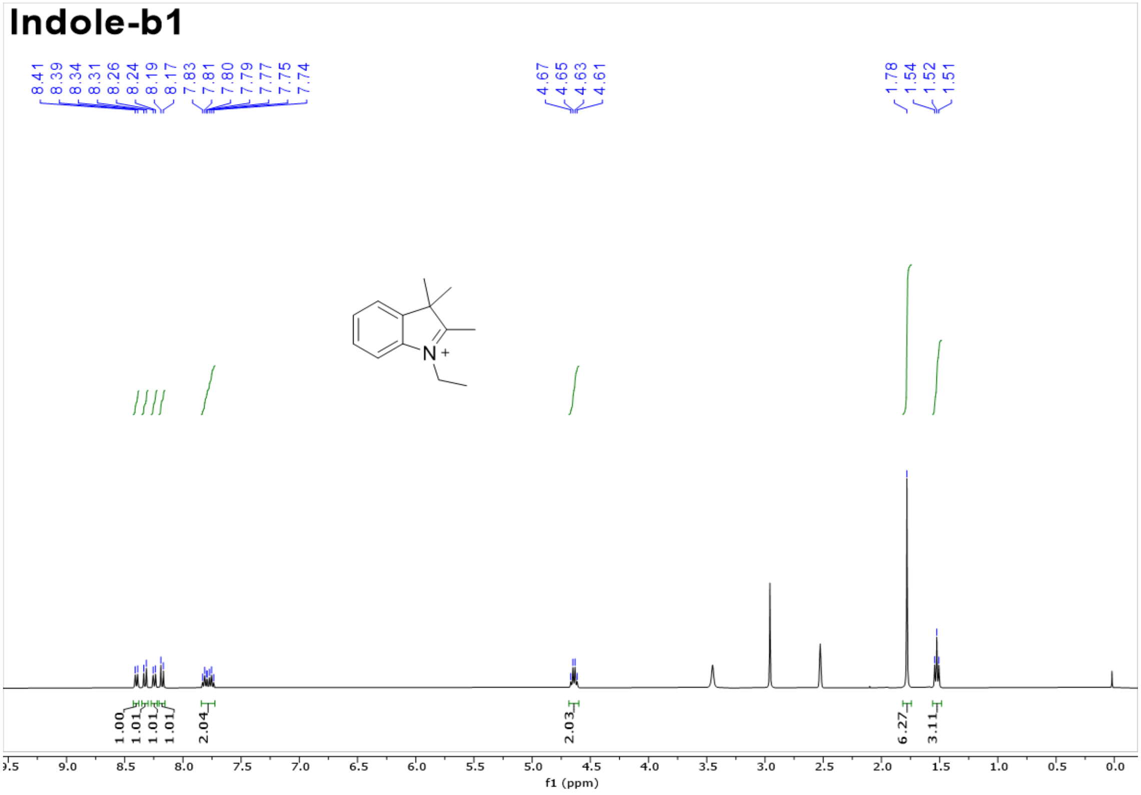

Indole-a2

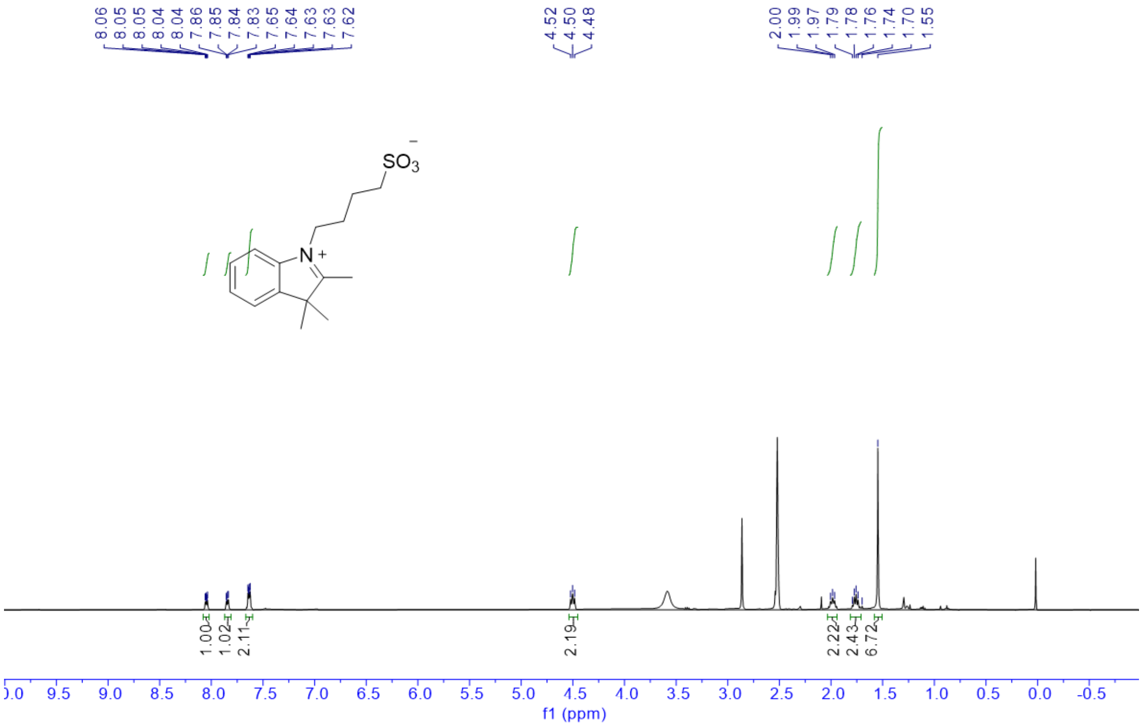

Indole-b2

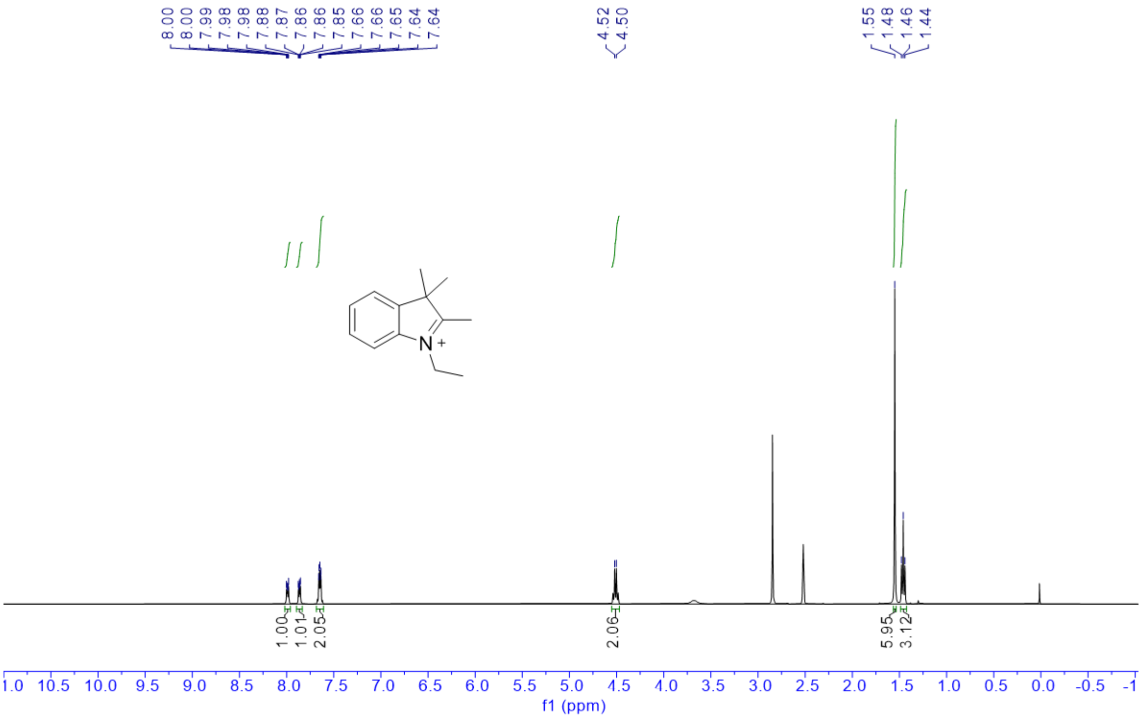

Indole-a3

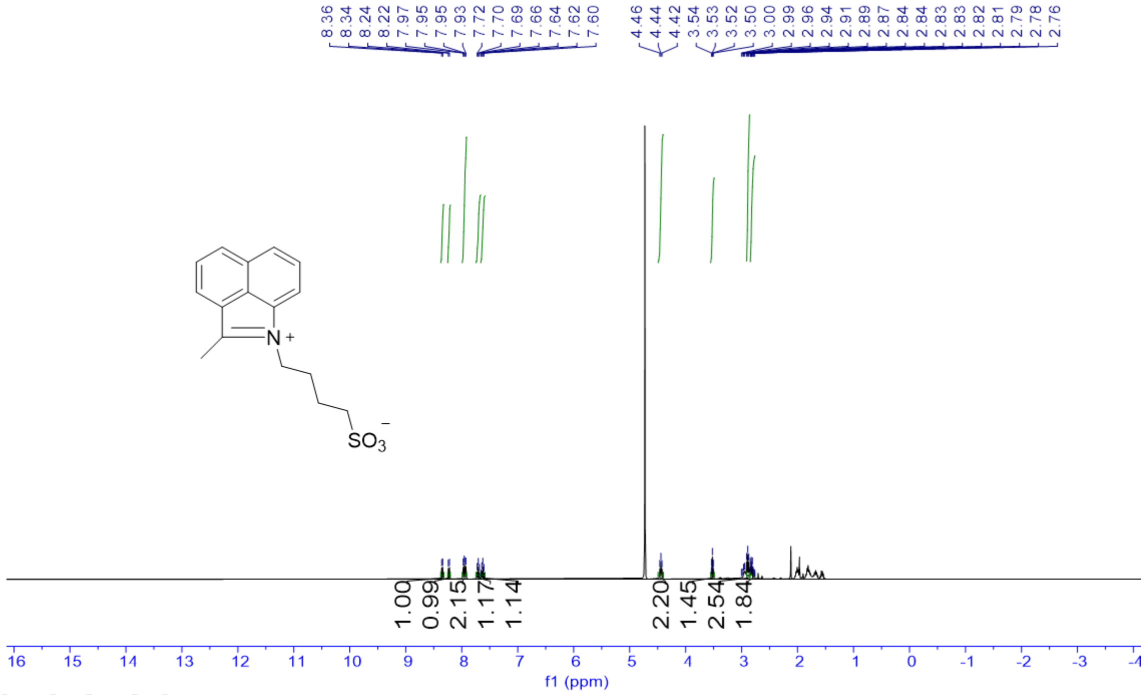

Indole-b3

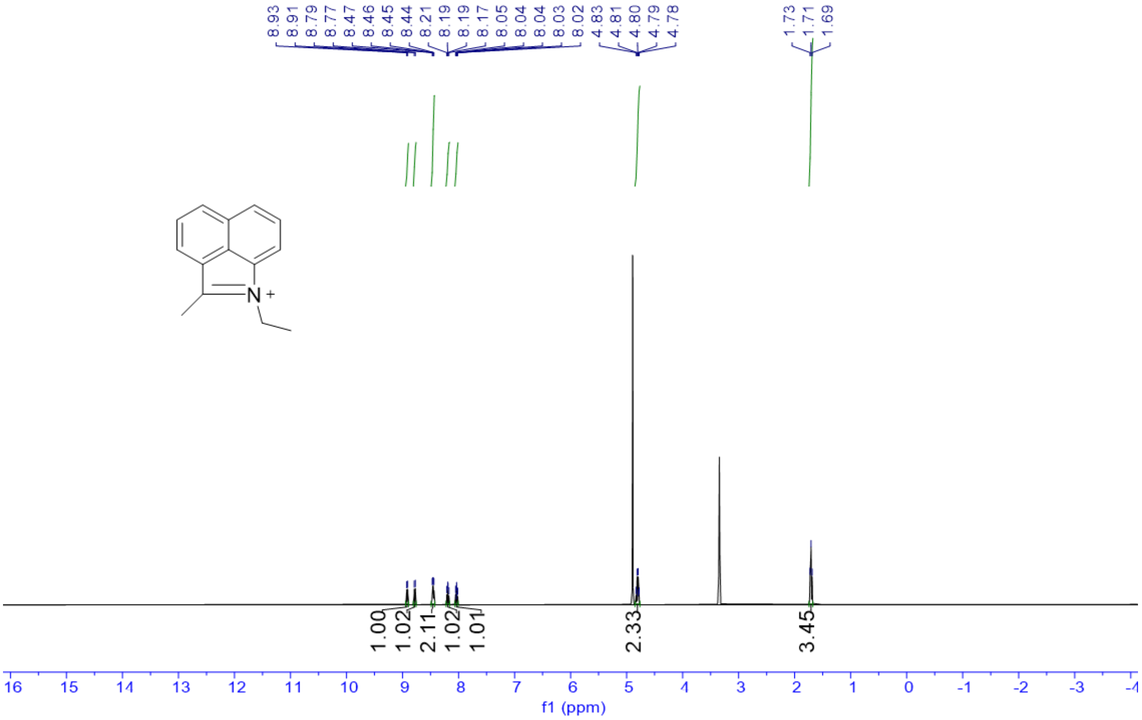

6. References

[1] S. Bhattacharya, R. S. Phatake, S. Nabha Barnea, N. Zerby, J. J. Zhu, R. Shikler, N. G. Lemcoff, R. Jelinek, *ACS Nano* **2019**, *13*, 1433-1442.

[2] Q. Yang, Z. Hu, S. Zhu, R. Ma, H. Ma, Z. Ma, H. Wan, T. Zhu, Z. Jiang, W. Liu, L. Jiao, H. Sun, Y. Liang, H. Dai, *J. Am. Chem. Soc.* **2018**, *140*, 1715-1724.

[3] a) H. Fan, W. Chen, J. Zhu, J. Zhang, S. Peng, *Int. Immunopharmacol* **2019**, *76*, 105909; b) Y. Tian, Y. Li, W. L. Jiang, D. Y. Zhou, J. Fei, C. Y. Li, *Anal. Chem.* **2019**, *91*, 10901-10907.

[4] T. Doi, K. Oikawa, J. Suzuki, M. Yoshida, N. Iki, *Synlett.* **2012**, *2012*, 306-310.

[5] S. Diao, J. L. Blackburn, G. Hong, A. L. Antaris, J. Chang, J. Z. Wu, B. Zhang, K. Cheng, C. J. Kuo, H. Dai, *Angew. Chem. Int. Ed.* **2015**, *54*, 14758-14762.

[6] S. Zhu, R. Tian, A. L. Antaris, X. Chen, H. Dai, *Adv. Mater.* **2019**, *31*, e1900321.

[7] a) S. J. Zhu, X. H. Zhao, Y. B. Song, S. Y. Lu, B. Yang, *Nano Today* **2016**, *11*, 128-132; b) S. Qu, X. Wang, Q. Lu, X. Liu, L. Wang, *Angew. Chem. Int. Ed.* **2012**, *51*, 12215-12218; c) K. Jiang, S. Sun, L. Zhang, Y. Lu, A. Wu, C. Cai, H. Lin, *Angew. Chem. Int. Ed.* **2015**, *54*, 5360-5363; d) S. Zhu, Q. Meng, L. Wang, J. Zhang, Y. Song, H. Jin, K. Zhang, H. Sun, H. Wang, B. Yang, *Angew. Chem. Int. Ed.* **2013**, *52*, 3953-3957; e) S. Tao, S. Zhu, T. Feng, C. Zheng, B. Yang, *Angew. Chem. Int. Ed.* **2020**, *59*, 9826-9840.

[8] Y. B. Shi, W. Yuan, Q. Y. Liu, M. Y. Kong, Z. H. Li, W. Feng, K. Hu, F. Y. Li, *ACS Mater. Lett.* **2019**, *1*, 418.
